# Endothelial HO-1 induction by model TG-rich lipoproteins is regulated through a NOX4-Nrf2 pathway^1^[S]

**DOI:** 10.1194/jlr.M067108

**Published:** 2016-07

**Authors:** Sally H. Latham Birt, Robert Purcell, Kathleen M. Botham, Caroline P. D. Wheeler-Jones

**Affiliations:** Department of Comparative Biomedical Sciences, Royal Veterinary College, London NW1 0TU, United Kingdom

**Keywords:** endothelial cells, lipids/oxidation, omega-3 fatty acids, cell signaling, fatty acid, artificial chylomicron remnant-like particles, heme oxygenase-1, reduced nicotinamide adenine dinucleotide phosphate oxidase 4, nuclear factor erythroid 2-related factor 2, triglyceride

## Abstract

Circulating levels of chylomicron remnants (CMRs) increase postprandially and their composition directly reflects dietary lipid intake. These TG-rich lipoproteins likely contribute to the development of endothelial dysfunction, albeit via unknown mechanisms. Here, we investigated how the FA composition of CMRs influences their actions on human aortic endothelial cells (HAECs) by comparing the effects of model CMRs—artificial TG-rich CMR-like particles (A-CRLPs)—containing TGs extracted from fish, DHA-rich algal, corn, or palm oils. HAECs responded with distinct transcriptional programs according to A-CRLP TG content and oxidation status, with genes involved in antioxidant defense and cytoprotection most prominently affected by *n*-3 PUFA-containing A-CRLPs. These particles were significantly more efficacious inducers of heme oxygenase-1 (HO-1) than *n*-6 PUFA corn or saturated FA-rich palm CRLPs. Mechanistically, HO-1 induction by all CRLPs requires NADPH oxidase 4, with PUFA-containing particles additionally dependent upon mitochondrial reactive oxygen species. Activation of both p38 MAPK and PPARβ/δ culminates in increased nuclear factor erythroid 2-related factor 2 (Nrf2) expression/nuclear translocation and HO-1 induction. These studies define new molecular pathways coupling endothelial cell activation by model CMRs with adaptive regulation of Nrf2-dependent HO-1 expression and may represent key mechanisms through which dietary FAs differentially impact progression of endothelial dysfunction.

Atherosclerosis and CVD are driven by endothelial dysfunction, a condition associated with low-grade systemic inflammation and characterized by inappropriate inflammatory activation of vascular endothelial cells (ECs). This disruption of endothelial homeostasis is coupled with impaired anti-inflammatory and antioxidant defense capacity, resulting in reduced resistance to oxidative and inflammatory insult, excessive reactive oxygen species (ROS) production, and development of oxidative stress (1). Thus, improving or maintaining endogenous mechanisms of endothelial protection may offer a strategy to mitigate development of CVD.

ECs are continuously challenged by the complex molecular milieu of circulating nutrients, cells, and blood constituents, including TG-rich lipoproteins (TRLs) [VLDLs, chylomicrons, and chylomicron remnants (CMRs)]. TRLs increase postprandially, but while endogenous lipoprotein involvement in CVD development is well-documented, how exogenous TRLs carrying dietary fats influence endothelial homeostasis is far less well understood, despite strong evidence implicating CMRs in the initiation of endothelial dysfunction (2–6). This is thought to involve a process now termed “postprandial inflammation” (7) in which CMRs migrate into the subendothelial space, activate circulating leukocytes, and increase immune cell trans-endothelial migration, potentially precipitating macrophage foam cell formation (3, 7).

The composition of CMRs directly reflects dietary lipid intake and, in individuals consuming a typical Western diet, ECs are chronically exposed to these TRLs for up to 18 h per day, so an improved understanding of their molecular actions on the endothelium is warranted. Studies using ex vivo postprandial TRL fractions and artificial model CMRs have demonstrated that these particles can trigger activation of MAPKs in ECs and influence EC inflammatory status (2, 8–12), although how these signaling pathways are coupled to altered gene expression and EC function is not defined. There are also suggestions that CMRs may exert protective or deleterious effects depending upon lipid content and pathophysiological context (3, 13), but the molecular details of their direct actions on human ECs remain elusive and the potential for divergent effects of CMRs relating to their specific FA content has not been addressed (3, 14). In this respect, we have shown that the FA composition of a high-fat test meal influences postprandial lipemia in healthy men and that *n*-3 PUFAs, particularly DHA, reduce plasma indices of oxidative stress when ingested as part of a high-fat meal (15), providing support for the purported relative beneficial actions of *n*-3 PUFAs in vivo (16). However, whether CMRs containing *n*-3 PUFAs impact vascular homeostasis through direct actions on ECs, if these effects differ from those of CMRs rich in other FAs, and how this operates at the molecular level have not been established.

Because ex vivo TRL fractions contain a complex mixture of both exogenous and endogenous lipoproteins, distinguishing the actions of VLDLs and their remnants from those of CMRs is challenging. Here, we have used our validated tractable model of ApoE-containing CMRs, “artificial TG-rich CMR-like particles” (A-CRLPs) (17), to establish whether they exert differential effects on primary human aortic ECs (HAECs) according to their FA composition, and have investigated the underlying molecular mechanisms. We have compared the effects of A-CRLPs containing TGs extracted from natural dietary oils relatively enriched in *n*-3 PUFAs, *n*-6 PUFAs, or saturated FAs (SFAs) to test the overarching hypothesis that model artificial CMRs carrying *n*-3 PUFAs exert greater cytoprotective effects than those rich in *n*-6 PUFAs or SFAs. This comparative analysis showed that A-CRLPs of varying FA content switch EC gene expression toward a program of antioxidant defense, dominated by induction of the nuclear factor erythroid 2-related factor 2 (Nrf2)-dependent gene, heme oxygenase-1 (HO-1), with *n*-3 PUFA-containing particles exhibiting the greatest efficacy. Our studies identify a new redox-dependent pathway through which model postprandial TRLs regulate EC gene expression and this may provide a molecular explanation for the relative beneficial effects of *n*-3 PUFAs in the postprandial phase.

## MATERIALS AND METHODS

Chemicals and reagents were from Sigma-Aldrich (Gillingham, UK) unless otherwise stated.

### Preparation and characterization of A-CRLPs

A-CRLPs containing ApoE were prepared as detailed previously (17). We and others have shown that these particles closely resemble physiological lipoproteins in terms of their size, density, and lipid composition and that they are cleared from the circulation in vivo in a manner that is identical to physiological particles (18–21). TGs were extracted from menhaden fish oil, DHASCO^®^ [a blend of DHA from *Crypthecodinium cohnii* with high oleic sunflower oil; now marketed as Life’s DHA (Martek Biosciences Corporation, Columbia, MD)], corn oil, and unrefined palm oil (KTC Edibles Ltd., UK) using Supelclean^TM^ LC-Diol SPE tubes. Oils were dissolved 1:10 in hexane, applied to preconditioned columns, and the flow-through discarded. TGs were eluted in 9:1 hexane:dichloromethane, assessed for purity by thin-layer chromatography, and stored under argon at −80°C (17).

Human plasma (National Blood Service, Colindale, UK) was prepared by ultracentrifugation to remove chylomicrons, CMRs, VLDLs, and IDLs and the remnant-free plasma stored at −20°C. A-CRLPs containing human ApoE were prepared with TGs from fish, DHASCO^®^, and corn and palm oils by ultracentrifugation, as detailed previously (17), using 70% TG, 2% cholesterol, 3% cholesteryl ester, and 25% phospholipids by weight. Briefly, 50 mg of emulsified lipids in 4.25 ml 25 mM Tricine (pH 7.4) were sonicated for 20 min at 55°C, 1.4 g KBr added, and samples overlaid with 2.5 ml 1.063 g/ml, 2.5 ml 1.020 g/ml, and 3 ml 1.006 g/ml saline prior to ultracentrifugation at 11,000 rpm for 20 min (20°C) in a SW40 Ti rotor (Beckman Coulter). The upper 1 ml was replaced with 1.006 g/ml NaCl and the tubes centrifuged at 23,500 rpm (1 h, 20°C). The top CRLP layer was mixed with 4 ml remnant-free plasma for 4 h at 37°C (for ApoE transfer), layered beneath 1.006 g/ml NaCl, centrifuged at 30,000 rpm for 16 h (12°C), and then washed with 1.006 g/ml NaCl by ultracentrifugation at 30,000 rpm for ≥3 h at 12°C. A remnant control was prepared in parallel using remnant-free plasma without lipids. The antioxidant compound, probucol (1 mg), was incorporated into separate preparations of fish, DHASCO^®^, and corn A-CRLPs to allow evaluation of their cellular effects when protected from oxidation. The total cholesterol and TG contents of A-CRLPs and probucol-containing A-CRLPs were determined using commercially available enzymatic assays (Infinity^TM^ cholesterol and Infinity^TM^ triglyceride reagents; ThermoFisher, UK) as per the manufacturer’s instructions, and their oxidation state assessed by measuring malondialdehyde using the thiobarbituric acid reactive substance (TBARS) assay, as described previously (17) (see supplementary Fig. 1).

### Cell culture and experimental incubations

HAECs were maintained in EGM-2 according to the supplier’s instructions (Lonza, Visp, Switzerland) and used for experiments at passages 6–8. Human umbilical vein endothelial cells (HUVECs) were isolated and cultured in M199 supplemented with 20% FCS and ECGS as previously described (22, 23), and used at passage 2. Prior to incubation with A-CRLPs, HAECs were serum-starved for ≥5 h in basal medium (EBM; Lonza) and HUVECs were serum-depleted for 1 h in 1% FBS-M199 (Gibco, ThermoFisher, UK) unless otherwise stated. Experimental treatments were prepared in basal medium except for those used in experiments measuring ROS production. A-CRLP incubations were at 280 μM TG for 4 or 16 h unless indicated otherwise.

### Microarray

Experimental details of a preliminary microarray performed in HAECs exposed to A-CRLPs of differing TG composition, together with its statistical analysis, are given in the supplementary file. The dataset has been placed in the GEO repository (record GSE80067; http://www.ncbi.nlm.nih.gov/geo/query/acc.cgi?token=alateuokffyxtgp&acc=GSE80067).

### ROS assays

Confluent ECs in 96-well plates were incubated with A-CRLPs for the times indicated in full growth medium without prior serum depletion. For incubations up to 1 h, cells were preloaded with dihydrorhodamine-1,2,3 (DHR; 10 μM) for 20 min before exposure to A-CRLPs. For longer incubations, ECs were challenged with A-CRLPs prior to addition of 10 μM DHR or 10 μM 2′7′-dichlorofluorescein-diacetate (DCF-DA) for 20 min, or 5 μM MitoSOX (ThermoFisher) for 10 min, as indicated. After washing in PBS, fluorescence was measured in a plate reader (Tecan, Switzerland) using excitation/emission wavelengths of 205/230 nm for DHR and DCF-DA, and 510/580 nm for MitoSOX.

### siRNA

HAECs in 6-well plates were cultured in EGM-2 until 50% confluent and washed with OptiMEM (Life Technologies, UK). Cells were transfected for 4 h with scrambled negative control siRNA or siRNAs targeting NADPH oxidase (NOX)4, p38^MAPKα^, PPARβ/δ, Akt1, or Akt2 (20 nM) complexed to ESCORT III in OptiMEM (see supplementary Table 1). Transfection media were replaced with EGM-2 and experimental incubations performed 48–72 h posttransfection.

### PCR

RNA was extracted using the GenElute™ mammalian total RNA miniprep kit according to the manufacturer’s instructions. Reverse transcription of 0.5 μg RNA per sample with 1 μM oligo dT primer (MWG Eurofins) and 1 U/ml RNaseOUT (Life Technologies; ThermoFisher) was performed with the OmniscriptRT kit (Qiagen, Manchester, UK) in a total reaction volume of 20 μl for 1 h (37°C). Real-time PCR using SYBR^®^ Green JumpStart™ Taq ReadyMix™ with MgCl_2_ was performed in a Chromo4 thermocycler (Bio-Rad, Hemel Hempstead, UK) using primers listed in supplementary Table 2. Data were processed using Opticon Monitor 3 software and normalized to the validated reference gene, GAPDH.

### Western blotting

Near confluent EC monolayers treated as indicated were washed with ice-cold PBS containing 0.4 mM Na_3_VO_4_ and then lysed in 76.5 mM Tris-HCl containing 10% (v/v) glycerol, 2% (w/v) SDS, 1 mM Na_3_VO_4_, and 10 μl/ml protease inhibitor cocktail on ice for 10 min. Proteins (30 μg) were resolved by SDS-PAGE (10%), transferred to polyvinylidene difluoride membranes, and immunoprobing was performed as described previously (24, 25). Membranes were blocked in 5% milk or 3% BSA, as appropriate, prior to incubation with primary antibodies (supplementary Table 3).

### Immunofluorescence microscopy

HUVECs on 1% gelatin-coated glass coverslips were treated as indicated, washed with PBS, fixed in paraformaldehyde (4%), permeabilized with 0.1% Triton X-100, and then blocked in 3% BSA/1% goat serum in PBS for 30 min. Coverslips were washed in PBS/0.1% Tween (three times for 5 min) and incubated with anti-Nrf2 antibody (1:200; Abcam, Cambridge, UK) diluted in 3% BSA/1% goat serum for 1 h. Following further washing, coverslips were incubated for 1 h with anti-rabbit Alexafluor 594 secondary antibody (1:1,000). Nuclei were stained with 4′,6-diamidino-2-phenylindole (DAPI) and images collected using an SP5 confocal microscope and Leica Application Suite advanced fluorescence software version 2.6 (Leica Microsystems, Milton Keynes, UK) with a 40× HCX PL FLUOTAR PH2 (numerical aperture = 0.75) objective lens. Mean Nrf2 intensity per DAPI-defined nucleus was quantified using Volocity^®^ 3D image analysis software (PerkinElmer, Waltham, MA).

### Statistical analysis

Data were collated in Microsoft Excel (MicroSoft, USA) with appropriate statistical analyses performed using GraphPad Prism (version 6.0; GraphPad Software) as detailed in the figure legends. Data are expressed as mean ± SEM and *P* < 0.05 was considered statistically significant.

## RESULTS

### A-CRLPs enriched in *n*-3 PUFAs induce HO-1 and reduce TXNIP and VCAM-1 expression to a greater extent than particles enriched in *n*-6 PUFAs or SFAs

Characterization of the model artificial TG-rich A-CRLPs used in this study confirmed that there was no significant variation in TG:cholesterol ratios between different preparations (supplementary Fig. 1) (17) and that their FA compositions were each reflective of the oil from which the TGs were extracted (not shown) (17). As anticipated, the *n*-3 PUFA-containing particles prepared using TGs from fish and DHASCO^®^ oils were more oxidized than those containing corn or palm oil TGs, and assessment of EC viability showed no deleterious effects of any of the A-CRLPs over a range of TG concentrations (supplementary Fig. 1). In normal healthy humans, the TG content of the postprandial CMR fraction at 5 h, corresponding to peak lipemia, approximates 300 μM (26); hence, A-CRLPs were used in subsequent experiments at the physiologically relevant concentration of 280 μM TG.

**Fig. 1.**
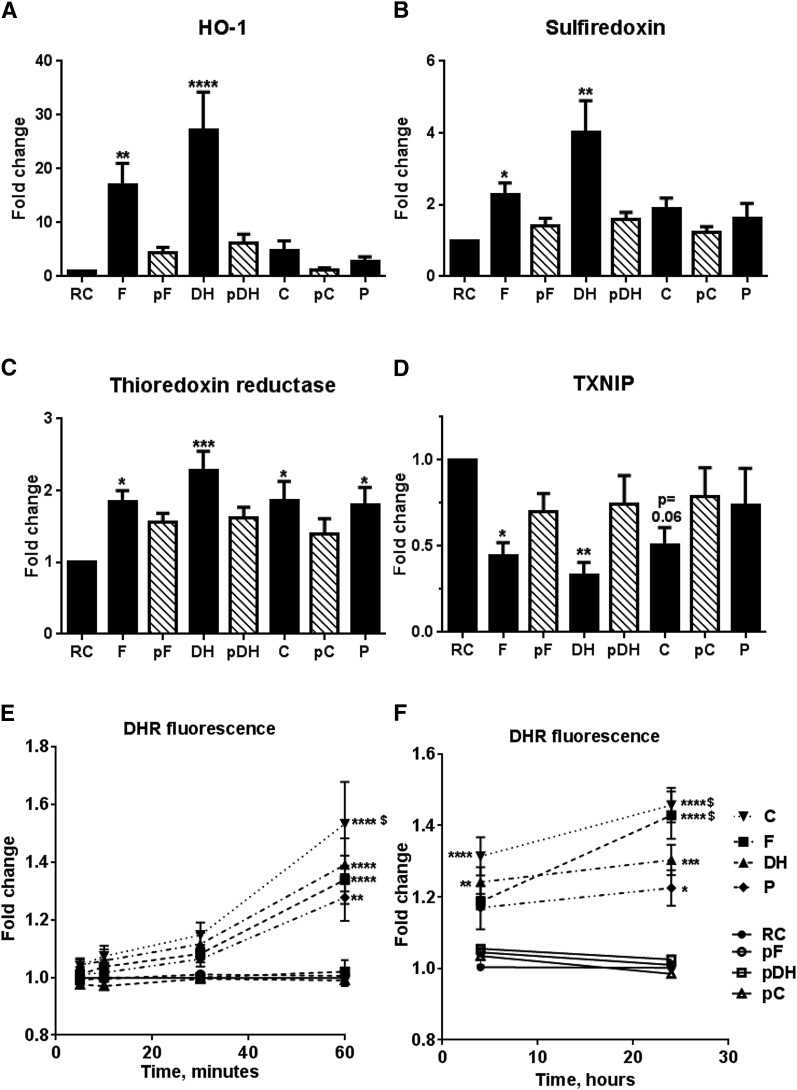
A-CRLPs modulate cytoprotective gene expression and elevate ROS production dependent upon oxidative state. A–D: HAECs were serum starved for 5 h and then incubated with A-CRLPs (280 μM TG) for 4 h. HO-1, SRX, TXR, and TXNIP mRNAs were quantified by qPCR and normalized to GAPDH (mean fold change versus RC ± SEM; n = 4–5. Black bars: remnant control (RC), fish (F), DHASCO^®^ (DH), corn (C), and palm (P) A-CRLPs. Striped bars: probucol-fish (pF), probucol-DHASCO^®^ (pDH), and probucol-corn (pC) A-CRLPs. One-way ANOVA with Dunnett’s multiple comparisons test, compared with RC: *P < 0.05, **P < 0.01, ****P < 0.0001. E, F: DHR was used to measure ROS/RNS production, presented as mean fold change relative to RC ± SEM (n = 6 for A-CRLPs, n = 3 for probucol-A-CRLPs with each experiment performed in technical quintuplet). Two-way ANOVA, RC versus A-CRLPs indicated: *P < 0.05, **P < 0.01, ***P < 0.001, ****P < 0.0001. For palm A-CRLPs versus other compositions, ^$^*P* < 0.05.

A preliminary microarray analysis assessed the comparative effects of A-CRLPs of differing FA composition on global HAEC gene expression (data and statistical analysis in supplementary Tables 4–7). Transcripts showing increased expression (>1.5-fold) 4 h after exposure to A-CRLPs included those encoding antioxidant proteins and protective molecules such as metallothioneins. Nrf2 target genes involved in antioxidant defense, such as HO-1, sulfiredoxin (SRX), and thioredoxin reductase (TXR), were particularly affected, with *n*-3 PUFA-containing A-CRLPs generally promoting greater fold induction than those enriched in *n*-6 PUFAs or SFAs. HO-1, for example, was upregulated by 6.84-fold (*P* = 0.004), 5.48-fold (*P* = 0.054), 3.78-fold (*P* = 0.0074), and 1.86-fold (*P* = 0.11) by fish, DHASCO^®^, corn, and palm A-CRLPs, respectively. Subsequent extensive and independent quantitative (q)PCR validation of selected genes identified by the array confirmed that fish and DHASCO^®^ A-CRLPs significantly increased expression of HO-1, SRX, and TXR mRNAs in HAECs after 4 h, with particularly robust effects on HO-1 expression (**Fig. 1A–C**), an antioxidant enzyme with cardiovascular protective properties (27–32). Corn and palm A-CRLPs promoted smaller, but nonetheless reproducible, increases in both HO-1 and SRX mRNAs (which did not reach statistical significance when analyzed alongside the marked effects of fish and DHASCO^®^ A-CRLPs) (Fig. 1A, B), and also increased TXR expression significantly (Fig. 1C). Of the transcripts identified by microarray as downregulated by A-CRLP treatment (supplementary Tables 4–7), thioredoxin interacting protein (TXNIP) was of particular interest because it acts as an endogenous inhibitor of thioredoxin by preventing its ability to scavenge ROS (33) and promotes inflammatory activation of ECs (34). Additional experiments confirmed that fish and DHASCO^®^ A-CRLPs significantly decreased TXNIP expression, with corn A-CRLPs showing a trend toward TXNIP reduction (*P* = 0.06) and SFA-containing A-CRLPs without effect (Fig. 1D). Incorporation of the antioxidant, probucol, which reduced the malondialdehyde content of PUFA-containing particles (supplementary Fig. 1), prevented A-CRLPs from significantly affecting gene expression, demonstrating that these actions depend upon A-CRLP oxidation state and/or their ability to undergo oxidation during experimental incubations (Fig. 1A–D). Given the known importance of ROS and reactive nitrogen species (RNS) in signal transduction, we then used the general probe, DHR, and showed that all A-CRLPs modestly, but significantly, increased ROS/RNS generation within 60 min, responses that were sustained for 24 h (Fig. 1E, F). As anticipated, PUFA-containing A-CRLPs increased ROS/RNS levels to a greater extent than SFA-rich palm A-CRLPs, while probucol-containing particles had no detectable effect (Fig. 1E, F). Together, these data imply that both TG composition and oxidative state are important determinants of the HAEC response to A-CRLPs.

**Fig. 2.**
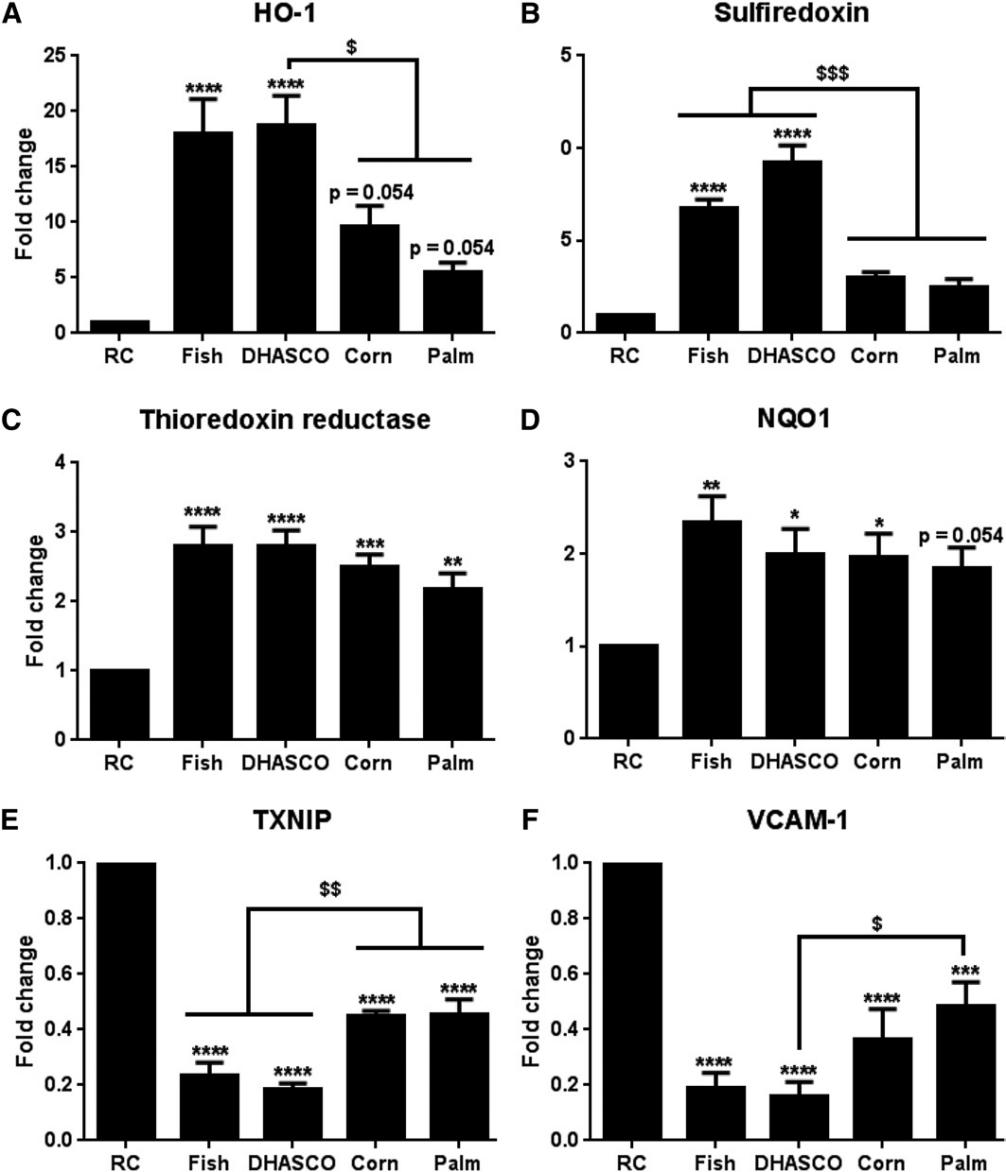
Differential effects of A-CRLPs with different FA compositions on gene expression in aortic ECs. HAECs were incubated with A-CRLPs (280 μM TG) for 16 h and mRNAs for the genes indicated in (A–F) measured by qPCR and normalized to GAPDH. Data are mean fold change from RC (±SEM; n = 5). One-way ANOVA with Tukey’s multiple comparisons test versus RC: *P < 0.05, **P < 0.01, ***P < 0.001, ****P < 0.0001 and compared with group(s) indicated: ^$^*P* < 0.05, ^$$^*P* < 0.01, ^$$$^*P* < 0.001.

Further studies demonstrated that the pattern of A-CRLP-induced expression of HO-1, SRX, and TXR in HAECs after 16 h was similar to that at 4 h (**Fig. 2A–C**) and that expression of NADPH quinone oxidoreductase-1, another well-characterized Nrf2 target gene (35) that was not induced at 4 h (not shown), was increased by more prolonged A-CRLP treatment (Fig. 2D). As at 4 h (Fig. 1), *n*-3 PUFA fish and DHASCO^®^ A-CRLPs showed significantly greater ability to induce HO-1 and SXR at 16 h than *n*-6 PUFA- or SFA-enriched A-CRLPs, with the latter showing a consistent trend toward HO-1 and SRX induction (albeit not statistically significant in direct comparison to the effects of *n*-3 PUFA-containing particles). Similarly, all particles reduced TXNIP mRNA after 16 h, but fish and DHASCO^®^ A-CRLPs did so to a significantly greater extent than corn and palm A-CRLPs (Fig. 2E). The effects of A-CRLPs on cytoprotective gene expression at 16 h were also accompanied by reduced levels of vascular cell adhesion molecule-1 (VCAM-1) (Fig. 2F), which, along with intracellular adhesion molecule-1, is important for supporting inflammatory changes in ECs (16, 36–38). Whereas A-CRLPs did not modify intracellular adhesion molecule-1 levels (not shown) VCAM-1 mRNA expression followed a similar pattern to that of TXNIP and was reduced by exposure to all CRLP types, with *n*-3 PUFA-containing DHASCO^®^ A-CRLPs causing a significantly greater inhibition than SFA-rich palm A-CRLPs (Fig. 2F). Thus, upregulation of Nrf2-regulated genes by A-CRLPs is associated with a concomitant downregulation in expression of genes with pro-inflammatory functions, and particles containing *n*-3 PUFAs appear to be more efficacious in these respects than those rich in SFAs.

### NOX4 activity and mitochondrial ROS regulate A-CRLP-induced HO-1 expression

**Fig. 3.**
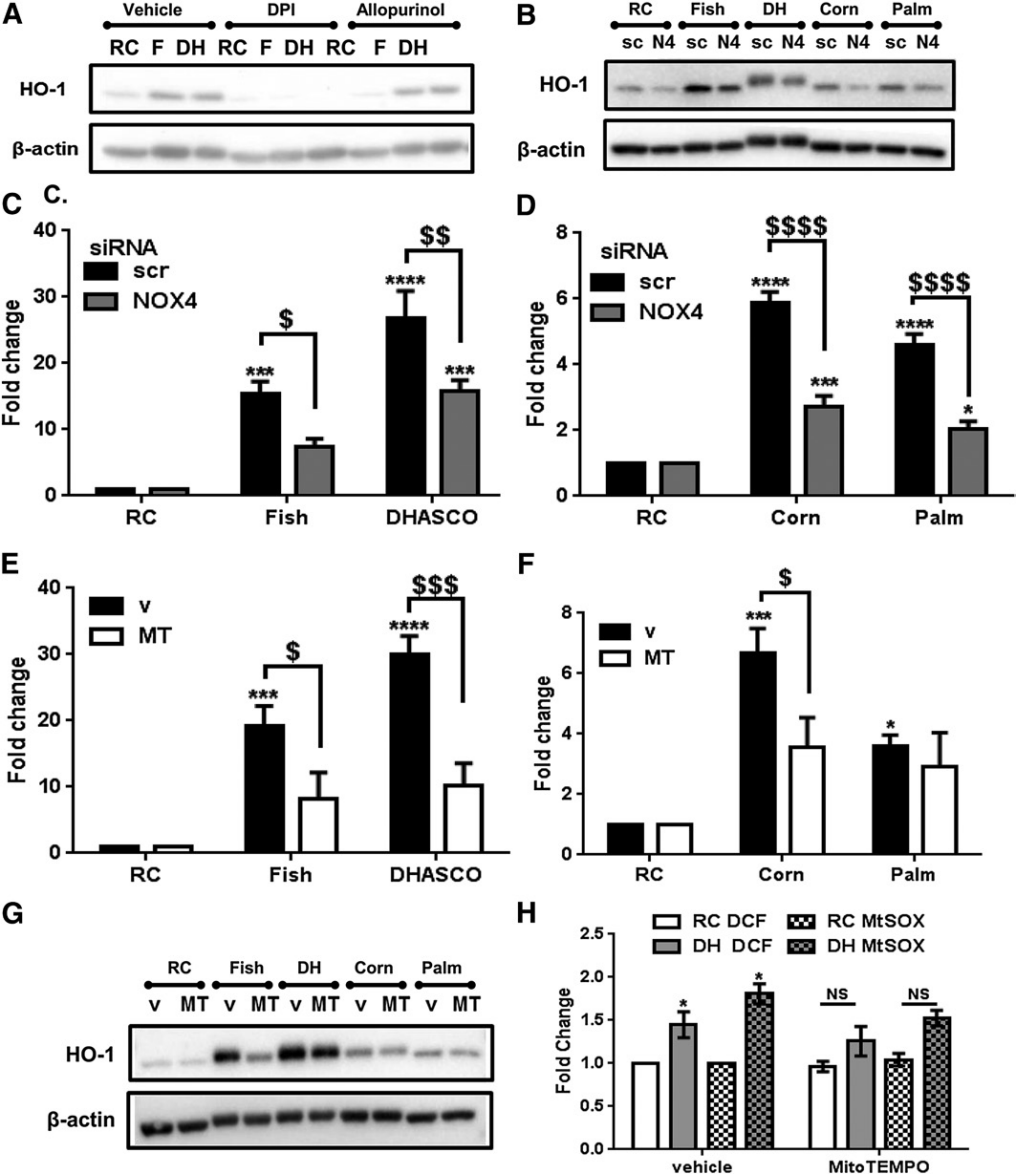
A-CRLP-driven HO-1 expression is controlled by NOX4 and mitochondrial ROS. A: After serum starvation, HAECs were pretreated with DMSO (vehicle), 5 μM diphenyleneiodonium, or 200 μM allopurinol for 1 h and then incubated with remnant control (RC), fish (F), or DHASCO^®^ (DH) A-CRLPs (280 μM TG) in the continued presence or absence of inhibitor for 4 h. Representative HO-1 and β-actin blots from three independent experiments are shown. B–D: HAECs transfected with scrambled (scr/sc) siRNA or NOX4 (N4) siRNA were serum-starved then incubated with A-CRLPs for 4 h. B: Representative Western blots from three independent experiments. C, D: qPCR for HO-1 mRNA normalized to GAPDH presented as fold change of RC, mean ± SEM (n = 3). Two-way ANOVA with Bonferroni’s multiple comparisons test, scr versus NOX4 siRNA; ^$^*P* < 0.05, ^$$^*P* < 0.01, ^$$$$^*P* < 0.0001. RC versus A-CRLPs: *P < 0.05, ***P < 0.001, ****P < 0.0001. E–G: Serum-starved HAECs were pretreated with DMSO (v) or 25 μM MitoTEMPO (MT) for 1 h then incubated with A-CRLPs for 4 h in the presence of DMSO or MT. E, F: qPCR for HO-1 mRNA normalized to GAPDH presented as fold change relative to RC, mean ± SEM, n = 3. Two-way ANOVA with Bonferroni’s multiple comparisons test, v versus MT; ^$^*P* < 0.05, ^$$$^*P* < 0.001. RC versus A-CRLP indicated; *P < 0.05, ***P < 0.001, ****P < 0.0001. G: Representative Western blots. H: HUVECs were pretreated with DMSO (vehicle) or 25 μM MitoTEMPO then incubated with RC or DHASCO^®^ (DH) A-CRLPs in the presence of vehicle/MitoTEMPO for 2 h prior to loading with the ROS probe, 2′7′-DCF-DA (DCF), or MitoSOX (MtSOX) as indicated. Data are mean fold change relative to RC ± SEM (n = 3 with each experiment performed in technical quintuplet). Two-way ANOVA with Bonferroni’s multiple comparisons test, RC versus DHASCO^®^; *P < 0.05.

Our data show that A-CRLPs induce antioxidant response gene expression in HAECs and increase ROS production, with probucol-containing particles corroborating the importance of oxidative state/ROS production for A-CRLP-driven gene expression. Because of the high level of HO-1 induction observed compared with that of the other validated genes, and the divergent effects of *n*-3 PUFA A-CRLPs on this versus the other particles (Figs. 1, 2), we focused our attention on delineating the mechanisms controlling HO-1 expression. First, we examined potential sources of the ROS produced in A-CRLP-treated ECs. Initial experiments in HUVECs showed that the antioxidant, *N*-acetyl cysteine, and the general flavoprotein inhibitor, diphenyleneiodonium, abrogated the effects of A-CRLPs on HO-1 expression, whereas apocynin had no effect at a concentration known to inhibit NOX2 (39) (supplementary Fig. 3). Using human monocyte cDNA as a positive control, HAECs were shown to express low/borderline detectable levels of NOX2 mRNA (not shown), agreeing with reports that endothelial NOX4 expression is ∼100-fold greater than that of NOX2 (40). Experiments in HAECs confirmed that diphenyleneiodonium abrogated A-CRLP-induced HO-1 expression in these cells, whereas the xanthine oxidase inhibitor, allopurinol, had no effect (**Fig. 3A**). These data collectively imply a predominant role for NOX4 in A-CRLP-stimulated HO-1 expression. In addition, DHASCO^®^ A-CRLPs reduced, while palm A-CRLPs increased, NOX4 mRNA after 16 h (supplementary Fig. 3), indicating divergent effects of sustained exposure to *n*-3 PUFA or SFA A-CRLPs on NOX4 expression.

To investigate the potential importance of NOX4 for regulating HO-1 induction by A-CRLPs, we used NOX4-targeted siRNA, which decreased endogenous NOX4 expression by ∼83% (supplementary Fig. 4A). These studies showed that NOX4 siRNA, but not noncoding scrambled siRNA, significantly reduced the ability of A-CRLPs to increase HO-1 expression at the mRNA level (Fig. 3C, D) with a similar trend observed at the protein level (Fig. 3B). NOX4 silencing did not completely abrogate the increased HO-1 levels evident in A-CRLP-stimulated cells, implying likely involvement of other mechanisms in this response. Accordingly, we used the mitochondrial superoxide scavenger, MitoTEMPO, to investigate the potential contribution of mitochondrial ROS generation. As shown in Fig. 3E–G, MitoTEMPO treatment reduced the ability of fish, DHASCO^®^, and corn A-CRLPs, but not palm A-CRLPs, to increase HO-1 expression, suggesting that mitochondrial ROS regulate HO-1 induction in response to PUFA-rich A-CRLPs, but not to SFA-rich palm A-CRLPs, which seem to depend primarily upon NOX4 activity for driving HO-1 induction. To verify that MitoTEMPO reduced A-CRLP-induced ROS production, we used the generic probe, DCF-DA, and the more specific superoxide indicator, MitoSOX. In vehicle (DMSO) preincubated cells, DHASCO^®^ A-CRLPs induced a significant increase in both DCF and MitoSOX fluorescence, but did not significantly increase either signal in cells preincubated with MitoTEMPO (Fig. 3H). Taken together, these data reveal a key role for NOX4 activity in regulating A-CRLP-stimulated HO-1 induction in ECs and indicate that both NOX4 activity and mitochondrial ROS contribute to HO-1 induction following exposure to A-CRLPs containing PUFAs.

### A-CRLP-induced HO-1 expression is dependent on Nrf2, but not on Akt or ERK1/2

**Fig. 4.**
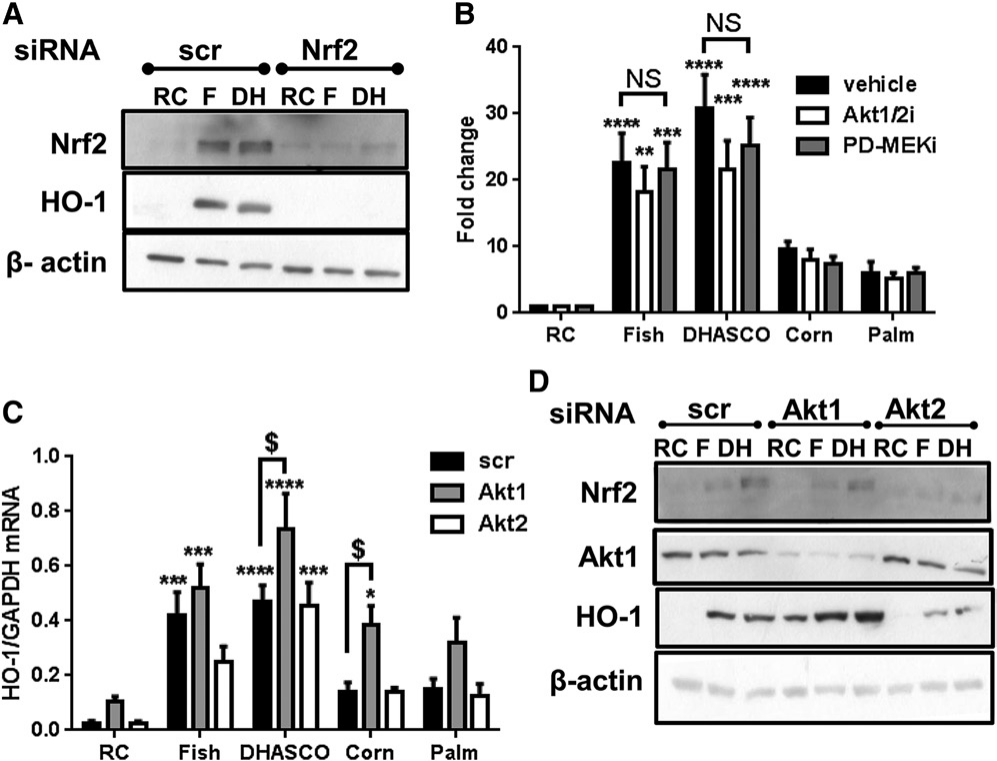
HO-1 induction by A-CRLPs is Nrf2 dependent but independent of Akt and ERK. A: HAECs transfected with 20 nM noncoding (scr) or Nrf2 siRNA were incubated with remnant control (RC), fish (F), or DHASCO^®^ (DH) A-CRLPs (280 μM TG) for 7 h. Representative blots from three independent experiments are shown. B: Serum-starved HAECs were preincubated with DMSO (vehicle), 1 μM Akt1/2i, or 1 μM PD184352 (PD-MEKi) for 1 h then incubated with A-CRLPs for 4 h in the continued presence or absence of inhibitor. HO-1 mRNA expression normalized to GAPDH is presented as fold change relative to RC (mean ± SEM, n = 3). Two-way ANOVA with Bonferroni’s multiple comparisons test, vehicle versus inhibitors not significant (NS). RC versus A-CRLP indicated: **P < 0.01, ***P < 0.001, ****P < 0.0001. C, D: HAECs transfected with 20 nM noncoding (scr), Akt1, or Akt2 siRNA were serum starved then incubated with A-CRLPs for 4 h. C: HO-1 mRNA normalized to GAPDH (fold change relative to RC; mean ± SEM, n = 4). Two-way ANOVA with Bonferroni’s multiple comparisons test, scr versus Akt1 siRNA: ^$^*P* < 0.05. RC versus CRLP: *P < 0.05, ***P < 0.001, ****P < 0.0001. D: Representative Western blots from four independent experiments are shown for Nrf2, Akt1, HO-1, and β-actin.

Having demonstrated the significance of ROS generation for HO-1 induction by A-CRLPs, we next evaluated the functional importance of Nrf2. As shown in **Fig. 4A**, transient transfection with siRNA directed against Nrf2, but not control siRNA, completely abrogated the increased HO-1 protein expression detected after incubation with fish or DHASCO^®^ A-CRLPs. In addition, while Nrf2 mRNA was unaffected by A-CRLPs (not shown), Nrf2 protein expression was increased in cells exposed to *n*-3 PUFA-containing particles, and attenuated by Nrf2 siRNA (Fig. 4A), consistent with a mechanism involving posttranslational stabilization of Nrf2 upon A-CRLP treatment. Treatment with NOX4 siRNA also reduced A-CRLP-induced Nrf2 expression (not shown), verifying NOX4’s importance as a regulator of Nrf2-dependent gene expression in ECs exposed to A-CRLPs.

Because other stimuli have been reported to induce HO-1 in HUVECs via Nrf2- and ROS-dependent mechanisms downstream of Akt or ERK (41), we determined whether similar pathways regulate HO-1 expression in aortic ECs challenged with A-CRLPs. Incubation with PUFA- or SFA-rich A-CRLPs increased phosphorylation of both Akt and ERK1/2 (supplementary Fig. 5). Next, we used a pharmacological inhibitor of Akt1 and Akt2 (Akt1/2i) and an inhibitor of MAPK kinase 1 (MEK; PD184352) to investigate whether Akt and/or the MEK/ERK pathway contribute to A-CRLP-induced HO-1 expression. At concentrations that abrogated A-CRLP-induced kinase phosphorylation (see supplementary Fig. 6), neither inhibitor significantly modulated A-CRLP-driven HO-1 mRNA expression (Fig. 4B), suggesting that these pathways are not coupled to altered HO-1 expression in HAECs. Reports of opposing functions of individual Akt isoforms (42) and the nonselectivity of Akt1/2i prompted us to investigate the relative involvement of Akt1 versus Akt2 using specific siRNAs targeting these isoforms (see supplementary Fig. 6A, B). Unexpectedly, Akt1 knockdown resulted in an ∼5-fold increase in basal HO-1 mRNA expression (supplementary Fig. 7) and this enhancement in expression was evident across all experimental samples at both mRNA and protein levels (Fig. 4C, D, respectively). Akt1 siRNA had no inhibitory effect on Nrf2 protein induction by *n*-3 PUFA-containing A-CRLPs (Fig. 4D) and silencing of Akt2, in contrast to Akt1, had no effect on basal HO-1 expression (supplementary Fig. 7), nor did it significantly affect A-CRLP-stimulated HO-1 induction (Fig. 4C). These data suggest that Akt1 operates as a negative regulator of HO-1 expression in aortic ECs, and additionally demonstrate that the stimulatory effects of A-CRLPs on Nrf2 and downstream HO-1 expression are independent of Akt1/2 activity.

### p38^MAPK^ and PPARβ/δ cooperatively regulate A-CRLP-induced HO-1 expression

**Fig. 5.**
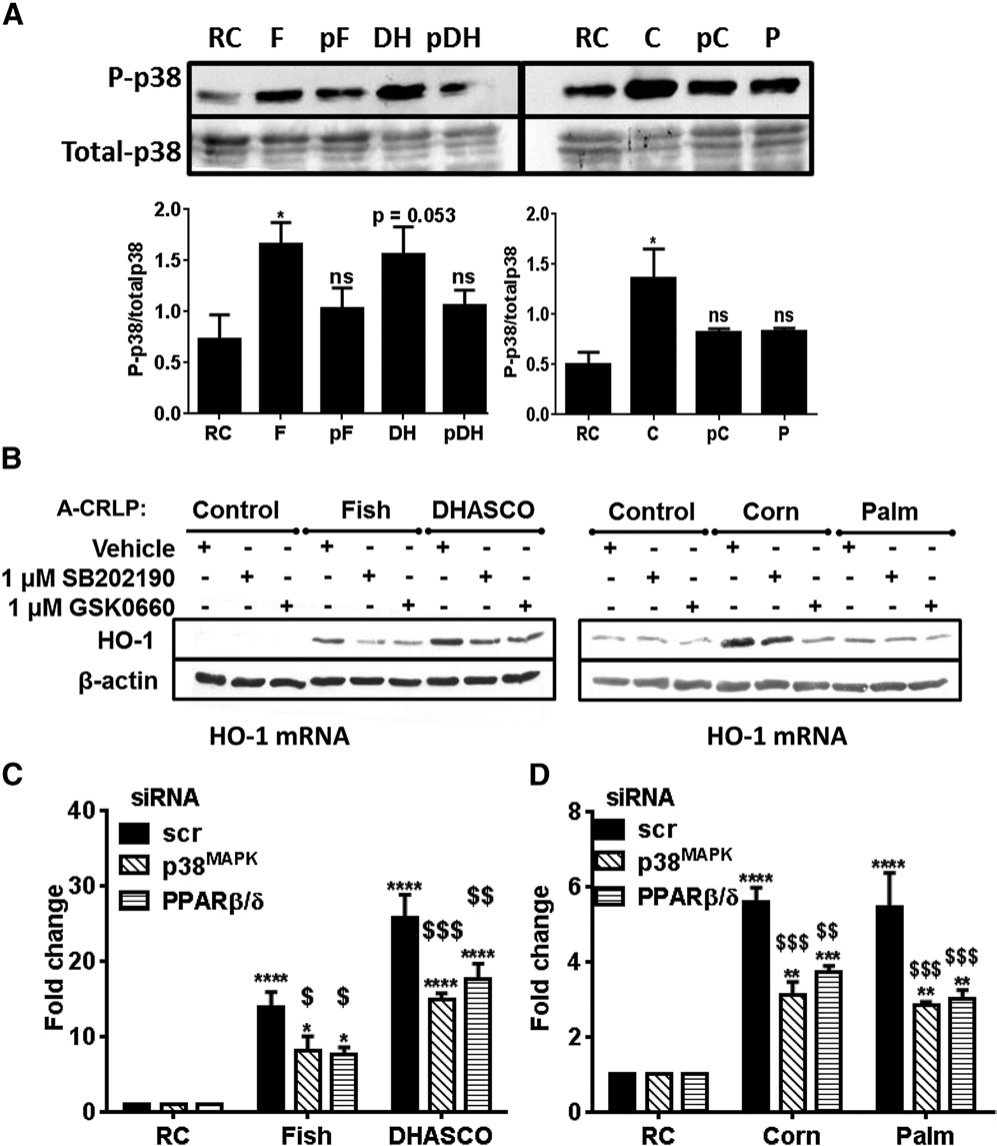
A-CRLP-induced HO-1 expression is regulated by p38^MAPK^ and PPARβ/δ. A: Serum-starved HAECs were incubated with remnant control (RC), fish (F), DHASCO^®^ (DH), probucol-DHASCO^®^ (pDH), corn (C), probucol-corn (pC), or palm (P) A-CRLPs at 280 μM TG for 10 min. Representative Western blots and densitometry analysis are shown for phospho- and total p38^MAPK^ (n = 3–4) *P < 0.05 versus RC. B: Serum-starved HAECs were pretreated with vehicle, 1 μM SB202190, or 1 μM GSK0660 for 1 h prior to a 4 h A-CRLP incubation in the absence/presence of inhibitor. Representative blots are shown (n = 3). C, D: HAECs were transfected with 20 nM noncoding (scr), MAPK14 (p38^MAPKα^), or PPARβ/δ siRNA and then incubated with A-CRLPs for 4 h. HO-1 mRNA normalized to GAPDH is given as fold change relative to RC (mean ± SEM, n = 3–4). Two-way ANOVA with Bonferroni’s multiple comparisons test, scr versus p38^MAPKα^ and scr versus PPARβ/δ as indicated: ^$^*P* < 0.05, ^$$^*P* < 0.01, ^$$$^*P* < 0.001. RC versus A-CRLP: **P < 0.01, ***P < 0.001, ****P < 0.0001.

We have previously shown that A-CRLPs trigger acute activation of p38 MAPK (p38^MAPK^) in HUVECs (8) and that p38^MAPK^ regulates beneficial EC functions, including proliferation and migration (24). While p38^MAPK^ can contribute to pro-inflammatory pathways upstream of nuclear factor-κB, there is also evidence linking p38^MAPK^ to Nrf2 activation in other cell types (43), but whether this pathway regulates Nrf2-responsive genes in ECs exposed to model dietary lipoproteins is not known. Similarly, pharmacological agonists of the lipid-sensing nuclear receptor, PPARβ/δ (44), can increase HO-1 expression (45, 46), but the role of endogenous PPARβ/δ in controlling HO-1 in A-CRLP-challenged ECs is undefined. Experiments in HAECs showed that PUFA-containing A-CRLPs significantly increased p38^MAPK^ phosphorylation and that this was reduced when the particles contained probucol (**Fig. 5A**), suggesting that activation of the p38^MAPK^ pathway is at least partly dependent upon the oxidation state of the particles. A-CRLPs containing SFAs also consistently increased p38^MAPK^ phosphorylation, although this did not quite reach significance following statistical analysis of combined densitometry data. Selective inhibitors of p38^MAPK^ (SB202190) and PPARβ/δ (GSK0660) were then used to examine involvement in A-CRLP-induced HO-1 induction. Neither SB202190 [which effectively reduced p38^MAPK^ phosphorylation (supplementary Fig. 6)] nor GSK0660 influenced basal HO-1 expression, but both inhibitors reduced A-CRLP-induced HO-1 induction at the protein (Fig. 5B) and mRNA levels (not shown). Next, we corroborated these findings using targeted siRNAs (see supplementary Fig. 4) and showed that knockdown of either p38^MAPKα^ (the principal functional isoform in ECs) or PPARβ/δ significantly reduced the increased HO-1 mRNA evident in cells exposed to A-CRLPs (Fig. 5C), confirming their importance as key regulators of A-CRLP-stimulated HO-1 expression.

**Fig. 6.**
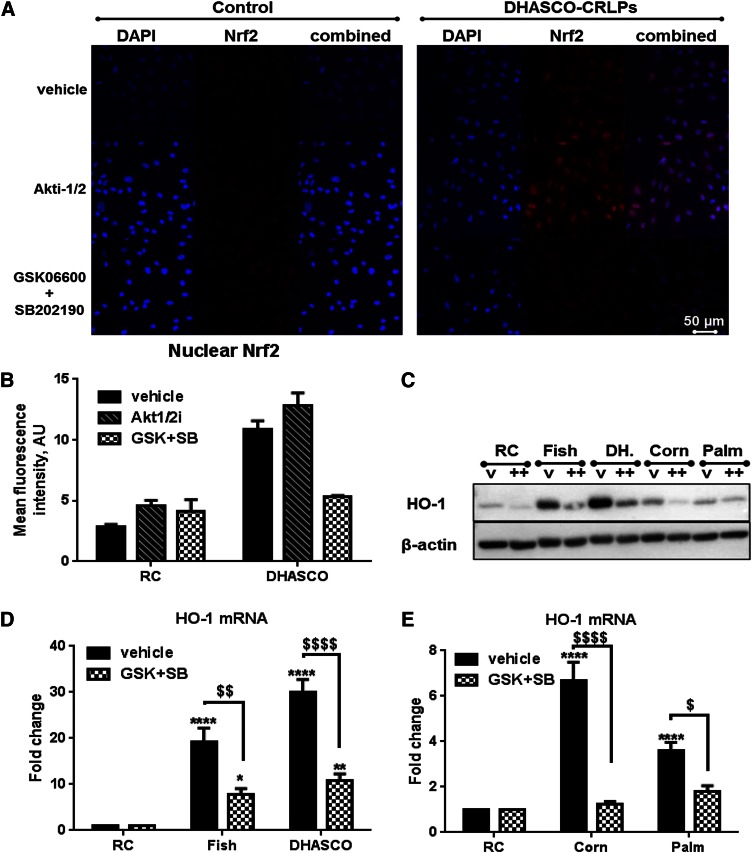
A-CRLPs increase Nrf2 expression and nuclear translocation via p38^MAPK^ and PPARβ/δ. A, B: ECs were serum depleted for 1 h, pretreated (1 h) with vehicle, 1 μM Akt1/2i, or both GSK0660 and SB202190 (each 1 μM) then incubated with remnant control (Control) or DHASCO^®^ A-CRLPs (280 μM TG) in the continued absence/presence of inhibitor(s) for 4 h. Cells were immunostained for Nrf2 (red) and nuclei labeled with DAPI (blue). No staining was evident in secondary antibody-only controls (not shown). Representative images are in (A) and combined mean nuclear Nrf2 fluorescence intensities from three separate images per condition are shown in (B) (mean ± SEM). C–E: Serum-starved HAECs were preincubated with vehicle or both 1 μM GSK0660 and 1 μM SB202190 (GSK+SB) for 1 h then incubated with A-CRLPs in the presence of vehicle or inhibitors for 4 h. C: Representative HO-1 and β-actin blots: vehicle (v), GSK+SB (++). D, E: HO-1 mRNA expression normalized to GAPDH. Data are fold change relative to remnant control (RC) (mean ± SEM, n = 3). Two-way ANOVA with Bonferroni’s multiple comparisons test: vehicle versus GSK+SB: ^$^*P* < 0.05, ^$$^*P* < 0.01, ^$$$$^*P* < 0.0001. RC versus A-CRLP: *P < 0.05, **P < 0.01, ****P < 0.0001.

To determine the relevance of p38^MAPK^ and PPARβ/δ activities proximal to Nrf2 activation, we used immunofluorescence to monitor Nrf2 in ECs exposed to DHASCO^®^ A-CRLPs (**Fig. 6A, B**). In keeping with the Nrf2-dependency of A-CRLP-induced Nrf2 and HO-1 expression (Fig. 4), *n*-3 PUFA-containing particles increased both expression and nuclear localization of Nrf2. These effects were not modified by pharmacological inhibition of Akt with Akt1/2i, confirming that increased Akt activity is not required for A-CRLP-stimulated Nrf2 activation, but were abolished by incubation with a combination of SB202190 and GSK0660 (Fig. 6A, B). Simultaneous inhibition of p38^MAPK^ and PPARβ/δ also resulted in near complete abrogation of HO-1 protein/mRNA induction by *n*-3 PUFA-containing A-CRLPs and total inhibition of the less robust HO-1 induction by A-CRLPs rich in *n*-6 PUFAs or SFAs (Fig. 6C–E). Together, these data demonstrate that p38^MAPK^ and PPARβ/δ cooperate to control Nrf2-dependent EC HO-1 expression in response to A-CRLPs.

## DISCUSSION

To our knowledge, this is the first detailed study exploring the effects of model CMRs on adult aortic ECs and provides novel mechanistic insight into how dietary lipoproteins of differing FA composition influence adult ECs. We show, using model A-CRLPs prepared using TGs extracted from four natural dietary oils, that A-CRLPs stimulate protective pathways in ECs and that particles containing *n*-3 PUFAs are particularly effective compared with those rich in either *n*-6 PUFAs or SFAs.

Nrf2 is a transcription factor that controls the adaptive response to endogenous and exogenous oxidative stress. Here, we identified several Nrf2-dependent genes modified by A-CRLPs, including antioxidant and cytoprotective molecules, demonstrating that modulation of Nrf2-regulated targets is one key EC response to lipoprotein challenge. HO-1 is a multifunctional enzyme, generating products with protective, anti-apoptotic, and anti-inflammatory actions (29, 32) and HO-1 deficiency is associated with accelerated endothelial dysfunction and atherosclerosis (27–32). Thus, the predominant and strong induction of HO-1 by *n*-3 PUFA-containing A-CRLPs, compared with those enriched in *n*-6 PUFAs or SFAs, suggests that a particularly robust antioxidant defense program may be triggered by EC exposure to TRL-associated *n*-3 PUFAs, changes that were mirrored by reduced expression of genes with known pro-inflammatory functions (VCAM-1 and TXNIP).

Silencing of Nrf2 confirmed its importance in A-CRLP-induced HO-1 induction. Also, Nrf2 protein was induced by *n*-3 PUFA-containing A-CRLPs, but we detected no change in Nrf2 mRNA, as reported for oxidant-induced Nrf2 (47), likely due to tight regulation of Nrf2 at the translational level via internal ribosome entry site-mediated translation and/or other mechanisms (48, 49). The relationship between enhanced HO-1 induction and the altered inflammatory potential identified here now requires exploration, as does efficacy in vivo, but it can be speculated that these endothelial-directed effects of *n*-3 PUFA-containing A-CRLPs may have mechanistic relevance for the actions of physiological TRLs in vivo and could help to explain the reported beneficial actions of dietary *n*-3 PUFAs on vascular homeostasis (3, 16). Indeed, in other functional studies directly comparing the EC-directed actions of A-CRLPs with those of physiological TRLs, we have established that CMR-rich TRL fractions isolated from humans following ingestion of high-fat meals supplemented with *n*-3 PUFAs (15) recapitulate the protective effects of *n*-3 PUFA A-CRLPs by upregulating antioxidant defense and have shown that HO-1 induction is linked to suppressed inflammation, notably reduced cytokine-stimulated adhesion molecule expression and monocyte adhesion to ECs, and improved EC survival (R. Purcell et al., unpublished observations). Nonesterified DHA has been reported to inhibit the modest increases in EC VCAM-1 expression elicited by “atherogenic” mixtures of TRLs isolated from hyperlipidemic individuals (50), but our study is the first demonstrating that a tractable artificial model of CMRs triggers protective responses in adult ECs according to FA content.

It is possible that the quantitatively dissimilar effects of the different A-CRLPs on HO-1 induction reflect the overall level of unsaturation of their constituent FAs, although this cannot be the full explanation, as *n*-6 PUFA corn A-CRLPs lead to equivalent ROS production to fish and DHASCO^®^ A-CRLPs (as detected using DHR), yet this did not correlate directly with their relative effects on HO-1 expression or their oxidative state indicated by TBARS content (*n*-3 PUFA A-CRLPs > *n*-6 PUFA A-CRLPs). Oxidation of the relatively *n*-6 PUFA-rich corn A-CRLPs may occur more slowly than the *n*-3 PUFA-containing particles, potentially explaining the difference in their TBARS contents. In addition, all A-CRLPs, including the SFA-rich particles, affected TXR and NADPH quinone oxidoreductase-1 expression to an equivalent extent after 16 h, despite the lower ability of SFA CRLPs to induce ROS compared with the other CRLPs. In keeping with their action to increase expression of cytoprotective genes, A-CRLPs had no detrimental effects on HAEC viability, so the ROS generated in response to these particles likely act as physiological signaling molecules rather than reflecting deleterious “oxidative stress” or lipid oxidation within ECs, cells which rely primarily on glycolysis and not on FA oxidation for energy (51). Probucol itself can also induce HO-1 independently of its antioxidant properties (52), which may explain the residual HO-1 induction by probucol-fish and -DHASCO^®^ A-CRLPs despite no detectable increase in ROS.

Together, our data demonstrate that both oxidation state and TG composition are important determinants of the EC response to model artificial CMRs. However, the lipid species and/or oxidation products mediating the differential effects of the A-CRLP types studied on EC gene expression have yet to be identified and are currently being investigated using lipidomics approaches. There are numerous potential candidates because, in addition to any direct effects of A-CRLP constituents (oxidized or otherwise) and of products of spontaneous oxidation in ECs, the activities of lipid-metabolizing enzymes, such as cyclooxygenases, lipoxygenases, and epoxygenases, operative in ECs, generate a plethora of oxidized FA metabolites that may contribute to the altered cell signaling and downstream gene expression described here. The specific oxidized lipid species contributing to the adaptive antioxidant/protective response triggered in ECs by *n*-3 PUFA-containing A-CRLPs are of particular interest. In this respect, it has been reported that oxidized DHA, itself, shows a greater ability to activate Nrf2 and induce NADPH quinone oxidoreductase-1 expression than parent DHA (53), suggesting that the more efficacious effects of *n*-3 PUFA-enriched A-CRLPs reported here could involve the actions of oxidized *n*-3 PUFAs. The residual HO-1 expression evident in ECs exposed to probucol-containing A-CRLPs also lends support to the notion that either the PUFAs themselves are partially responsible and/or that lipid oxidation occurs at the cell surface/within ECs following delivery of the PUFAs in TG form via the lipoproteins.

A combination of pharmacological and siRNA approaches strongly implicated NOX4, highly expressed by ECs (40, 54), as a driver of HO-1 expression by A-CRLPs. Interestingly, NOX4 mRNA expression was reduced by DHASCO^®^ A-CRLPs and increased by palm CRLPs, in keeping with previous studies demonstrating that nonesterified DHA decreases, whereas palmitate increases, NOX4 (55, 56) and suggesting that redox regulatory feedback mechanisms involving altered NOX4 expression may be operating in a TG-dependent manner in ECs. MitoTEMPO, a mitochondrial superoxide scavenger, also reduced A-CRLP-stimulated HO-1 expression, most significantly for *n*-3 PUFA-rich particles, with no effect on the response to palm A-CRLPs. Thus, our data suggest that NOX4 activity and mitochondrial ROS regulate HO-1 induction by A-CRLPs, with SFA-rich palm A-CRLPs inducing HO-1 primarily via NOX4 and *n*-3 PUFA-rich A-CRLPs using both mechanisms to upregulate endothelial HO-1. While it is known that inappropriate expression of NOX4 can lead to redox imbalance and pathology (57), the NOX4/Nrf2-dependency of protective HO-1 induction by A-CRLPs reported here is wholly consistent with recent evidence in murine models that NOX4, when specifically coupled to Nrf2 activation, protects the heart during chronic pressure overload (58) and that endothelial NOX4 provides endogenous protection from atherosclerosis development (59).

We showed that HAECs exposed to A-CRLPs exhibited enhanced phosphorylation of Akt, ERK1/2, and p38^MAPK^, implicating these kinases in the control of ECs by dietary FAs. Given the known pro-survival actions of the Akt and MEK-ERK signaling pathways, we hypothesized that these might be key mediators of the antioxidant defense responses triggered by A-CRLPs. However, although Akt1 appears to regulate basal HO-1 expression, we found that p38^MAPK^ is the dominant pathway coupled to downstream regulation of A-CRLP-driven Nrf2-dependent HO-1 expression, consistent with a growing body of evidence linking p38^MAPK^ activation to cell survival and oxidative stress resistance (24, 43, 60–62). The significance of the A-CRLP-induced increases in Akt and ERK1/2 activities remains to be determined, but these could be important for transcriptional regulation of other gene targets identified in A-CRLP-treated HAECs and, thus, for the functional outcomes of A-CRLP exposure, which are currently under investigation.

PPARβ/δ is a lipid ligand-inducible transcription factor with established roles in the regulation of lipid metabolism and endothelial function (44, 63). Although there is poor understanding of its specific involvement in nutrient sensing by ECs and subsequent alterations in gene expression, there is evidence that a wide range of FAs can directly activate PPARβ/δ (64, 65). Here, PPARβ/δ-deficient HAECs showed a blunted response to A-CRLPs, demonstrating that PPARβ/δ is a pivotal mediator of A-CRLP-stimulated HO-1 induction in adult ECs. The precise mechanisms leading to PPARβ/δ activation require clarification but, as discussed above, FAs and/or their oxidation products delivered to ECs by A-CRLPs or subsequently generated by enzymatic processes within ECs are likely to directly activate PPARβ/δ. Given that PUFAs are more potent ligands of PPARβ/δ than SFAs (44), our finding that PPARβ/δ regulates HO-1 induction by A-CRLPs irrespective of their FA composition may reflect the fact that TGs extracted from the four natural oils are complex mixtures of FAs present within the model artificial CMRs, alongside cholesterol, esterified cholesterol, and phospholipids. Our observation that PPARβ/δ plays a role in HO-1 induction by A-CRLPs is consistent with reports that endothelial-specific PPARβ/δ deletion in mice is associated with reduced expression of protective genes and heightened inflammation (66), and that direct pharmacological activation of PPARβ/δ increases HO-1 expression in fetal ECs and murine aorta ex vivo (46, 67).

The modest stimulation of HO-1 induction by SFA-rich palm CRLPs, together with their lack of effect on cell viability, suggest that these particles are not overtly deleterious to ECs, contrasting with reports that nonesterified palmitate alone drives lipotoxicity and EC dysfunction in vitro (68). However, it is noteworthy that HO-1 induction in response to SFA A-CRLPs was accompanied both by increased expression of E-selectin (detected in the array; validation not shown) and by enhanced NOX4 expression after more prolonged incubation. Thus, while some common cytoprotective pathways appeared to be triggered by all the A-CRLP types studied, albeit to different extents, other divergent effects occurring in parallel will likely dictate the overall outcome of endothelial exposure to particles with distinct TG compositions.

**Fig. 7.**
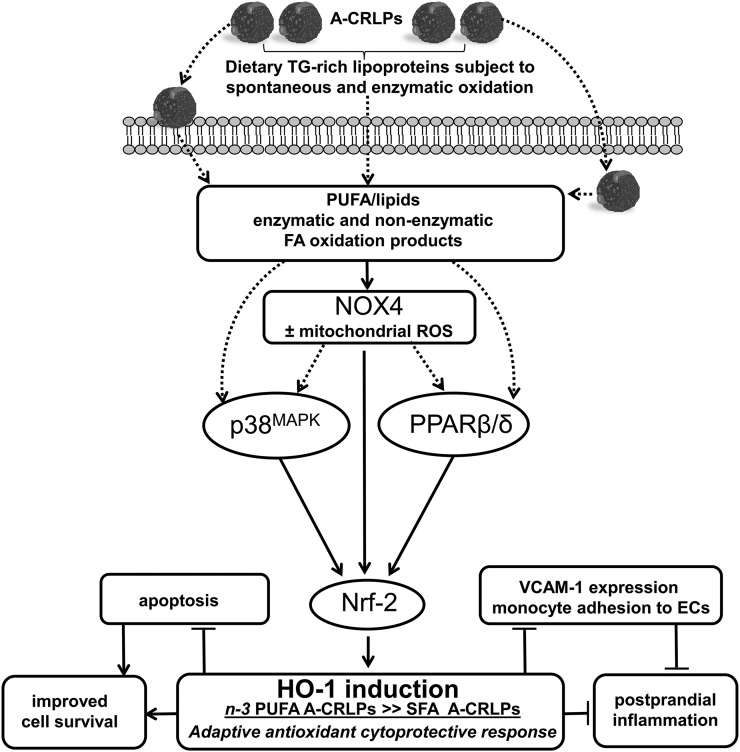
Summary of results: proposed mechanisms mediating the effects of A-CRLPs on HAECs. Artificial model TRLs resembling CMRs (A-CRLPs) upregulate several antioxidant and cytoprotective genes in HAECs with the most efficacious effects evident with A-CRLPs containing TG extracted from *n*-3 PUFA-containing oils. A-CRLPs regulate HO-1 induction in HAECs through a NOX4-driven pathway involving downstream activation of p38^MAPK^ and parallel activation of the nuclear receptor superfamily member, PPARβ/δ. This results in increased Nrf2 expression and nuclear translocation, culminating in transcriptional upregulation of HO-1 and of other protective/antioxidant genes (e.g., SRX and TXR). We also showed that A-CRLP-driven HO-1 induction in HAECs is accompanied by reduced expression of the pro-inflammatory adhesion molecule, VCAM-1. The lipid species mediating activation of this redox-dependent pathway are yet to be clarified, but it is reasonable to speculate that oxidized FA/lipids acting directly on ECs following metabolism of TG-rich A-CRLPs at the cell surface and/or enzymatic products of FA/lipid oxidation generated intracellularly following particle uptake play key roles. Because HO-1 has known anti-inflammatory and cytoprotective functions, TRLs carrying *n*-3 PUFAs may act to suppress inflammatory changes in the endothelium and maintain cell survival in the postprandial phase, thus moderating the postprandial inflammation associated with fat consumption.

Collectively, our data are consistent with a mechanism in which A-CRLP treatment results in activation of NOX4 leading to redox-dependent activation of p38^MAPK^ and parallel activation of the lipid sensor, PPARβ/δ (see **Fig. 7**). This results in increased Nrf2 expression and activity and transcriptional regulation of HO-1 and other protective genes. Activation of a NOX4-Nrf2-HO-1 axis triggered by dietary-derived TRLs in vivo may, therefore, be fundamentally important for adaptive protection of the vessel wall in the postprandial phase and for mitigating the postprandial inflammation thought to encourage development of endothelial dysfunction and its detrimental consequences (3).

## Supplementary Material

Supplemental Data
