# Supplementary material for: Endothelial HO-1 induction by model TG-rich lipoproteins is regulated through a NOX4-Nrf2 pathway
Source: J Lipid Res. 2016 Jul;57(7):1204–18. doi: 10.1194/jlr.M067108 (PMC4918850; doi:10.1194/jlr.M067108)
Supplement: Supplemental Data [file supp_57_7_1204__index.html]

Endothelial HO-1 induction by model TG-rich lipoproteins is regulated through a NOX4-Nrf2 pathway — Endothelial HO-1 induction by model TG-rich lipoproteins is regulated through a NOX4-Nrf2 pathway — Supplemental Data 

# Endothelial HO-1 induction by model TG-rich lipoproteins is regulated through a NOX4-Nrf2 pathway

## Supplemental Data

- Supplementary information (.pdf, 770 KB) - Supplemental methods, tables and figures
