# Supplementary material for: Endothelial HO-1 induction by model TG-rich lipoproteins is regulated through a NOX4-Nrf2 pathway
Source: J Lipid Res. 2016 Jul;57(7):1204–18. doi: 10.1194/jlr.M067108 (PMC4918850; doi:10.1194/jlr.M067108)
Supplement: Supplemental Data [file 10.1194_M067108_jlr.M067108-1.pdf]

Supplementary Information

Methods

Microarray

4 individual isolates of near-confluent HAECs in 6cm dishes were serum-starved overnight (in endothelial basal medium) and each isolate incubated with an individually prepared batch of A-CRLPs for 4 hours as follows: 1, remnant control (RC); 2-5, A-CRLPs (280 µM TG) containing TG extracted from fish, DHASCO, corn and palm oils, respectively; 6, an additional PBS control.

RNA was extracted using the RNeasy mini kit (Qiagen, Manchester, UK) and stored at -80 ºC until analysis. RNA samples were characterized using a Nanodrop 1000 (concentration and purity verification) and Aligent 2100 Bioanalyzer to verify RNA integrity (RIN scores >9). The Illumina TotalPrep™ RNA Amplification kit (Ambion®, ThermoFisher, UK) was used according to the manufacturer's instructions to synthesize labelled cRNA, of which 750 ng per sample was hybridized to Illumina HumanHT-12_4_BeadChip arrays in a pseudorandomized manner. RNA hybridization and chip scanning were performed according to the manufacturer's instructions, and image data were imported into GenomeStudio (Illumina, San Diego, CA). Missing values were imputed, and non-normalised data and quantile normalised data were exported from GenomeStudio according to standard operating procedures at St. George's Medical Biomics Centre (University of London). The complete data set can be accessed from the NCBI GEO repository, record GSE80067.

Fold changes were calculated for each A-CRLP-treated sample, and the additional PBS control, relative to RC for the 4 different HAEC isolates. Transcripts of mean fold change >1.5 or <0.7 were considered as potentially differentially regulated transcripts/genes for further investigation. There were no such differentially regulated genes detected between the PBS and RC controls (not shown). *P*-values were calculated using paired two-tailed Student's t-Tests. Multiple comparisons were corrected for using the two-stage linear step-up procedure of Benjamini, Krieger and Yekutieli (Prism software), with the false discovery rate (FDR) or "Q" set to 5%.

Supplementary Table 1: siRNA details

Target details (supplier)		Sequence(s) 5’-3’
“scr”<br>non-coding<br>control	ON-TARGETplus Non-targeting siRNA pool<br>D-001810-01-05 (5 nmol) lot # 1634172, sequences not available<br>(GE Healthcare Dharmacon Inc., Lafayette, CO, United States)	
Nrf2	siRNA against Nrf 2, target: 5’-AAGAGTATGAGCTGGAAAAAC-3’<br>Custom siRNA 1027020 (QIAGEN)	
Akt1	ON-TARGETplus SMARTpool®<br>Human AKT1 (207) siRNA<br>L-003000-00-0005<br>(Dharmacon)	CAUCACACCACCUGACCAA<br>ACAAGGACGGGCACAUUAA<br>CAAGGGCACUUUCGGCAAG<br>UCACAGCCCUGAAGUACUC
Akt2	ON-TARGETplus SMARTpool®<br>Human AKT2 siRNA<br>(Dharmacon)	Sequences not available<br>Product code L-003001-00-0005<br>Lot number 100323
NOX4	ON-TARGETplus SMARTpool®<br>Human NOX4 (50507) siRNA<br>L-010194-00-0005<br>(Dharmacon)	ACUAUGAUAUCUUCUGGUA<br>GAAAUUAUCCCAAGCUGUA<br>GGGCUAGGAUUGUGUCUAA<br>GAUCACAGCCUCUACAUAU
p38$^{MAPK\alpha}$<br>(MAPK14)	ON-TARGETplus SMARTpool® Human<br>MAPK14 (1432) siRNA<br>L-003512-00-0005<br>(Dharmacon)	GGAAUUCAAUGAUGUGUAU<br>UCUCCGAGGUCUAAAGUAU<br>GUAAUCUAGCUGUGAAUGA<br>GUCCAUCAUUCAUGCGAAA
PPARβ/δ<br>(PPARD)	ON-TARGETplus SMARTpool® Human<br>PPARD (5467) siRNA<br>L-003435-00-0005<br>(Dharmacon)	GACCUGGCCCUAUUCAUUG<br>UAUCAUUGAGCCUAAGUUU<br>GAGCGCAGCUGCAACAUUC<br>CCACUACGGUGUUCAUGCA

Supplementary Table 2: Primers for qPCR

Target	Forward primer 5’-3’	Reverse primer 5’-3’
GAPDH	GTCATCCATGACAACTTT GGTATC	AGTAGAGGCAGGGATGATGTTCT
HO-1	AGCAACAAAGTGCAAGATTCTG	CATAAAGCCCTACAGCAACTGTC
SRX	CCAGGGAGGTGACTACTTCTACTC	CTCCCAGGTACACCCTTAGGTCT
TXR	TGGGACAGAATGATAGAAGCTGTA	TTATTTGTTGCCTTAATCCTGTGA
TXNIP	GCAGTGCAAACAGACTTCGG	TCACCTGTTGGCTGGTCTTC
VCAM-1	AAGATGGTCGTGATCCTTGG	GGTGCTGCAAGTCAATGAGA
NQO1	CCTGGAAGGATGGAAGAAACG	AGAATCCTGCCTGGAAGTTTAGG
Nrf2	AAGCCATTCACTCTCTGAACTTCT	CTGGGACTTGTGTTTAGTGAAATG
Akt1	TCGTGTGGCAGCACGTGTACG	TCAGTCTCCGACGTGACCTGG
Akt2	TCAACTGGCAGGACGTGGTCC	ACCTTGTGTCGACCTCGGACG
NOX4	CAGCAAGATACCGAGATGAGG	GTAGAGGCTGTGATCATGAGGAATAG
$p38^{MAPK}\alpha$	GGGGGCAGATCTGAACAACA	CAACAGCTCGGCCATTATGC
PPARβ/δ	CATCGAGACATTGTGGCAGG	GCACGCCATACTTGAGAAGG

Supplementary Table 3: Antibodies

Target	Catalog #	Supplier	Dilution
β-actin	A5316	Sigma-Aldrich, Gillingham, UK	1:5000
HO-1	SPA-896	Stressgen, Enzo Life Sciences, Exeter, UK	1:4000
Nrf2	Ab62352	Abcam, Cambridge, UK	1:1000
Akt1	2938	Cell Signaling Technology Danvers, MA, United States	1:1000
P-Akt Ser 473	4060	Cell Signaling Technology	1:1000
Pan-Akt	2920	Cell Signaling Technology	1:1000
P-ERK Thr 202/ Tyr 204	9101	Cell Signaling Technology	1:1000
ERK1	SC-94	Santa Cruz, Insight Biotechnology Limited, Wembley, UK	1:5000
P-p38^{MAPK} Thr180/Tyr182	4511	Cell Signaling Technology	1:1000
Total p38^{MAPK}	9212	Cell Signaling Technology	1:1000
Anti-Rabbit IgG - HRP	31460	Pierce, ThermoFisher UK	1:50000
Anti-Mouse IgG - HRP	31430	Pierce, ThermoFisher UK	1:50000

Supplementary Table 4. Fish A-CRLPs microarray data. Data analysis: multiple paired two-tailed Student's t-Tests, multiple comparison correction by two-stage linear step-up procedure of Benjamini, Krieger and Yekutieli. **Statistically significant findings are shown in bold (FDR 5%).**

**Transcripts up-regulated >1.5 fold by Fish A-CRLPs**				
**Illumina ID**	**Gene Name**	**Mean Fold Change**	***P* value**	**q value**
**ILMN_1800512**	**heme oxygenase 1**	**6.84**	**0.004**	**0.024**
**ILMN_1795963**	**oxidative stress induced growth inhibitor 1 (previously OKL38)**	**3.22**	**0.000**	**0.003**
**ILMN_2319588**		**1.73**	**0.011**	**0.037**
**ILMN_1707727**	**angiopoietin-like 4**	**2.63**	**0.014**	**0.042**
**ILMN_1788547**	**glutamate-cysteine ligase, modifier subunit**	**2.50**	**0.002**	**0.016**
**ILMN_2225974**		**1.96**	**0.001**	**0.012**
ILMN_2184373	C-X-C motif chemokine ligand 8 (previously interleukin 8)	2.50	0.146	0.135
ILMN_1666733		2.20	0.154	0.135
ILMN_1738335	tumor necrosis factor superfamily member 18	2.21	0.048	0.064
ILMN_1804822	sulfiredoxin 1	2.12	0.026	0.052
ILMN_3210914	PREDICTED: misc_RNA (LOC344887) miscRNA	1.93	0.032	0.052
ILMN_1740426	ras related dexamethasone induced 1	1.88	0.060	0.075
ILMN_1775170	metallothionein 1X	1.84	0.136	0.135

**ILMN_1717056**	**thioredoxin reductase 1**	**1.83**	**0.009**	**0.034**
**ILMN_2324421**		**1.65**	**0.016**	**0.044**
**ILMN_1700232**	**F-box protein 30**	**1.81**	**0.002**	**0.016**
**ILMN_2168992**		**1.64**	**0.017**	**0.045**
ILMN_1657435	metallothionein 1M	1.72	0.185	0.152
ILMN_1718766	metallothionein 1F	1.69	0.159	0.135
**ILMN_1763627**	**transportin 1**	**1.62**	**0.020**	**0.046**
ILMN_1655229	solute carrier family 7 member 11	1.56	0.029	0.052
ILMN_2089875	tumor necrosis factor superfamily member 4	1.52	0.160	0.135
ILMN_2186061	6-phosphofructo-2-kinase/fructose-2,6-biphosphatase 3	1.52	0.031	0.052
ILMN_1769245	GLI pathogenesis-related 1	1.51	0.035	0.054
**Transcripts down-regulated <0.7 fold by Fish A-CRLPs**				
ILMN_3243644	PREDICTED: hypothetical protein LOC100132564	0.47	0.059	0.075
ILMN_3251587	RNA, 28S ribosomal 5	0.53	0.084	0.094
**ILMN_1656111**	**myosin regulatory light chain interacting protein**	**0.54**	**0.009**	**0.034**
**ILMN_1697448**	**thioredoxin interacting protein**	**0.55**	**0.007**	**0.034**
ILMN_1733559	RNA, 28S ribosomal 5	0.58	0.039	0.057

ILMN_1663080	LFNG O-fucosylpeptide 3-beta-N-acetylglucosaminyltransferase	0.64	0.033	0.052
ILMN_3246805	PREDICTED: hypothetical protein LOC100134364	0.64	0.094	0.098
ILMN_3241034	small nucleolar RNA, C/D box 3C	0.64	0.042	0.059
ILMN_3249578	PREDICTED: hypothetical protein LOC100132394	0.64	0.075	0.090
ILMN_3239574	small nucleolar RNA, C/D box 3A	0.66	0.107	0.109
**ILMN_2082865**	**plasmolipin**	**0.67**	**0.001**	**0.014**
ILMN_1703852	ephrin B2	0.67	0.080	0.093
ILMN_3242315	small nucleolar RNA, C/D box 3D	0.67	0.159	0.135
**ILMN_1770641**	**kelch like family member 3**	**0.68**	**0.020**	**0.046**
ILMN_1745079	tripartite motif containing 2	0.68	0.027	0.052
ILMN_3240220	RNA, U1 small nuclear 4 (previously RNU1F1)	0.69	0.094	0.098
ILMN_1766054	ATP binding cassette subfamily A member 1	0.70	0.222	0.177

Supplementary Table 5. DHASCO A-CRLPs microarray data (not statistically significant).

Illumina ID	Gene Name	Mean Fold Change	*P*-value
**Transcripts up-regulated >1.5 fold by DHASCO A-CRLPs**			
ILMN_1800512	heme oxygenase 1	5.48	0.0537
ILMN_2184373	C-X-C motif chemokine ligand 8 (interleukin 8)	2.61	0.0695
ILMN_1666733		2.12	0.0955
ILMN_1795963	oxidative stress induced growth inhibitor 1 (OKL38)	2.28	0.0159
ILMN_1707727	angiopoietin-like 4	2.01	0.0178
ILMN_1740426	ras related dexamethasone induced 1	1.89	0.2228
ILMN_1788547	glutamate-cysteine ligase, modifier subunit	1.83	0.0677
ILMN_2225974		1.56	0.0832
ILMN_2186061	6-phosphofructo-2-kinase/fructose-2,6-biphosphatase 3	1.69	0.1395
ILMN_1797728	3-hydroxy-3-methylglutaryl-Coenzyme A synthase 1	1.65	0.0144
ILMN_1738335	tumor necrosis factor superfamily member 18	1.56	0.0265
ILMN_2168992	F-box protein 30	1.51	0.0756
ILMN_3210914	PREDICTED: misc_RNA (LOC344887) miscRNA	1.51	0.1454
**Transcripts down-regulated <0.7 fold by DHASCO A-CRLPs**			
ILMN_1656111	myosin regulatory light chain interacting protein	0.55	0.0044

ILMN_1697448	thioredoxin interacting protein	0.56	0.0044
ILMN_3243644	PREDICTED: hypothetical protein LOC100132564	0.56	0.0901
ILMN_3251587	RNA, 28S ribosomal 5	0.62	0.1195
ILMN_1663080	LFNG O-fucosylpeptide 3-beta-N-acetylglucosaminyltransferase	0.62	0.0268
ILMN_1703852	ephrin B2	0.63	0.0798
ILMN_3308138	RNA, U4 small nuclear 2	0.65	0.1101
ILMN_1739423	RNA, 7SK small nuclear	0.68	0.1366
ILMN_3242315	small nucleolar RNA, C/D box 3D	0.68	0.1226
ILMN_3241034	small nucleolar RNA, C/D box 3C	0.69	0.0173
ILMN_1766054	ATP binding cassette subfamily A member 1	0.70	0.2178

Supplementary Table 6. Corn A-CRLPs microarray data (not statistically significant)

Transcripts up-regulated >1.5 fold by Corn A-CRLPs			
**Illumina ID**	**Gene Name**	**Mean Fold Change**	***P*-value**
ILMN_1800512	heme oxygenase 1	3.78	0.007
ILMN_2184373	C-X-C motif chemokine ligand 8 (interleukin 8)	2.76	0.126
ILMN_1666733		2.22	0.196
ILMN_1795963	oxidative stress induced growth inhibitor 1 (OKL38)	2.28	0.022
ILMN_1707727	angiopoietin-like 4	2.11	0.031
ILMN_1740426	ras related dexamethasone induced 1	1.96	0.179
ILMN_1739393	selectin E	1.87	0.174
ILMN_2186061	6-phosphofructo-2-kinase/fructose-2,6-biphosphatase 3	1.74	0.042
ILMN_1788547	glutamate-cysteine ligase, modifier subunit	1.68	0.035
ILMN_2225974		1.56	0.036
ILMN_1738335	tumor necrosis factor superfamily member 18	1.68	0.022
ILMN_1797728	3-hydroxy-3-methylglutaryl-Coenzyme A synthase 1	1.67	0.112
ILMN_1718766	metallothionein 1F	1.67	0.140
ILMN_1717056	thioredoxin reductase 1	1.65	0.035
ILMN_2324421		1.55	0.081

ILMN_1700081	follistatin	1.59	0.046
ILMN_1711838	solute carrier family 25 member 24	1.58	0.057
ILMN_1683859	solute carrier family 7 member 1	1.56	0.048
ILMN_1775170	metallothionein 1X	1.56	0.137
ILMN_1720373	solute carrier family 7 member 5	1.55	0.016
ILMN_1804822	sulfiredoxin 1	1.54	0.106
ILMN_3305938	serum/glucocorticoid regulated kinase 1	1.52	0.069
ILMN_1657435	metallothionein 1M	1.52	0.148
ILMN_1677607	sterol-C5-desaturase	1.52	0.027
ILMN_1746175	tumor necrosis factor superfamily member 4	1.51	0.253
ILMN_3210914	PREDICTED: misc_RNA (LOC344887) miscRNA	1.51	0.032
ILMN_1722718	bone morphogenetic protein 2	1.51	0.036
**Transcripts down-regulated <0.7 fold by Corn A-CRLPs**			
ILMN_3243644	PREDICTED: hypothetical protein LOC100132564	0.50	0.063
ILMN_1656111	myosin regulatory light chain interacting protein	0.53	0.008
ILMN_3242315	small nucleolar RNA, C/D box 3D	0.55	0.089
ILMN_3251587	RNA, 28S ribosomal 5	0.58	0.088
ILMN_1733559		0.63	0.057
ILMN_3241034	small nucleolar RNA, C/D box 3C	0.59	0.026

ILMN_1739423	RNA, 7SK small nuclear	0.60	0.080
ILMN_2074860		0.66	0.160
ILMN_1697448	thioredoxin interacting protein	0.60	0.005
ILMN_3246805	PREDICTED: hypothetical protein LOC100134364	0.61	0.073
ILMN_3239574	small nucleolar RNA, C/D box 3A	0.62	0.081
ILMN_3249578	PREDICTED: hypothetical protein LOC100132394	0.63	0.069
ILMN_3244646	RNA, U1 small nuclear 4	0.64	0.115
ILMN_3240220	(previously RNU1G2 and RNU1F1)	0.64	0.045
ILMN_1703852	ephrin B2	0.64	0.074
ILMN_1732988	RIMS binding protein 3 (previously KIAA1666)	0.66	0.109
ILMN_3245678	RNA, U1 small nuclear 1 (previously RNU1A3)	0.67	0.112
ILMN_1663080	LFNG O-fucosylpeptide 3-beta-N-acetylglucosaminyltransferase	0.67	0.018
ILMN_3308138	RNA, U4 small nuclear 2	0.67	0.149
ILMN_1794782	ATP binding cassette subfamily G member 1	0.68	0.049
ILMN_3246273	RNA, U1 small nuclear 3	0.69	0.189

Supplementary Table 7. Palm A-CRLPs microarray data (not statistically significant)

Transcripts up-regulated >1.5 fold by Palm A-CRLPs			
**Illumina ID**	**Gene Name**	**Mean Fold Change**	***P*-value**
ILMN_1739393	selectin E	2.62	0.164
ILMN_2184373	C-X-C motif chemokine ligand 8 (interleukin 8)	2.49	0.196
ILMN_1666733		2.38	0.130
ILMN_1775170	metallothionein 1X	2.35	0.203
ILMN_1657435	metallothionein 1M	2.16	0.190
ILMN_1718766	metallothionein 1F	2.06	0.139
ILMN_1800512	heme oxygenase 1	1.86	0.113
ILMN_1738335	tumor necrosis factor superfamily member 18	1.83	0.175
ILMN_1715401	metallothionein 1G	1.71	0.231
ILMN_1709348	aldehyde dehydrogenase 1 family member A1	1.70	0.073
ILMN_1740426	ras related dexamethasone induced 1	1.67	0.418
ILMN_1707727	angiopoietin-like 4	1.67	0.043
ILMN_1769245	GLI pathogenesis-related 1	1.66	0.013
ILMN_2092536	heat shock protein family E (Hsp10) member 1	1.57	0.359
ILMN_1781400	solute carrier family 7 member 2	1.57	0.194
ILMN_2071809	matrix Gla protein	1.57	0.091

Transcripts down-regulated <0.7 fold by Palm A-CRLPs			
ILMN_1733559	RNA, 28S ribosomal 5	0.56	0.040
LMN_3243644	PREDICTED: hypothetical protein LOC100132564	0.56	0.100
ILMN_1656111	myosin regulatory light chain interacting protein	0.64	0.022
ILMN_3251587	RNA, 28S ribosomal 5	0.65	0.185
ILMN_3240220	RNA, U1 small nuclear 4 (previously RNU1F1)	0.66	0.026
ILMN_3246805	PREDICTED: hypothetical protein LOC100134364	0.67	0.164
ILMN_1697448	thioredoxin interacting protein	0.67	0.017
ILMN_1794782	ATP binding cassette subfamily G member 1	0.69	0.133
ILMN_3249578	PREDICTED: hypothetical protein LOC100132394	0.69	0.155
ILMN_1703852	ephrin B2	0.69	0.095

Footnote to Supplementary Tables 4-7: In supplementary Tables 4-7, cells shaded grey indicate that the predicted hypothetical protein record indicated has been withdrawn by NCBI because the model on which it was based was not predicted in a later annotation. However, several such transcripts were detected (as down-regulated by A-CRLP incubations) and these findings may indicate that the sequences in question are from transcripts that have an important, as yet unknown, non-coding function.

Supplementary Figure 1

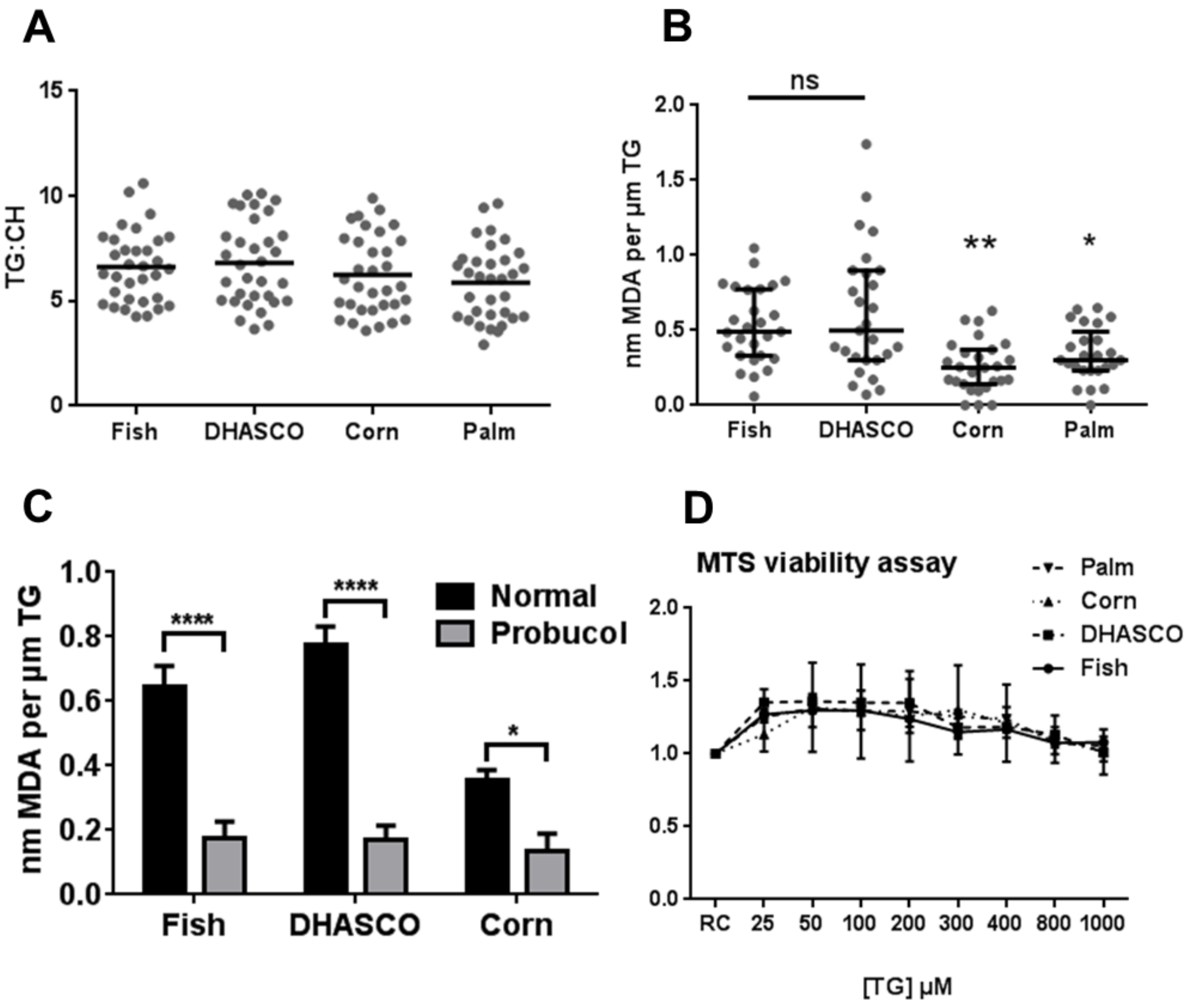

Supplementary Figure 1: Characterization of A-CRLPs

A. TG:cholesterol (CH) ratios of the 4 A-CRLP types; n = 33 separate preparations, mean ratio indicated. **B-C.** TBARS data presented as nmol malondialdehyde (MDA) per µmol TG. **B:** median and interquartile range indicated; n = 25 individual preparations. One-way ANOVA with Dunnett's multiple comparisons test, compared to fish A-CRLPs: **P* < 0.05, ***P* < 0.01. **C:** mean data comparing normal and probucol-containing A-CRLPs, n =3, two way ANOVA with Sidak's multiple comparisons test: *$P < 0.05$, ****$P < 0.0001$ **D**. HAEC incubated with A-CRLPs (concentrations indicated) for 24 hours were treated with CellTiter 96® AQueous One Solution Cell Proliferation Assay (Promega, Southampton, UK) as per the manufacturer's instructions to estimate viability. Data are presented as mean fold change from remnant control (RC) ± SEM; n = 4 independent experiments each performed in technical triplicate.

Supplementary Figure 2

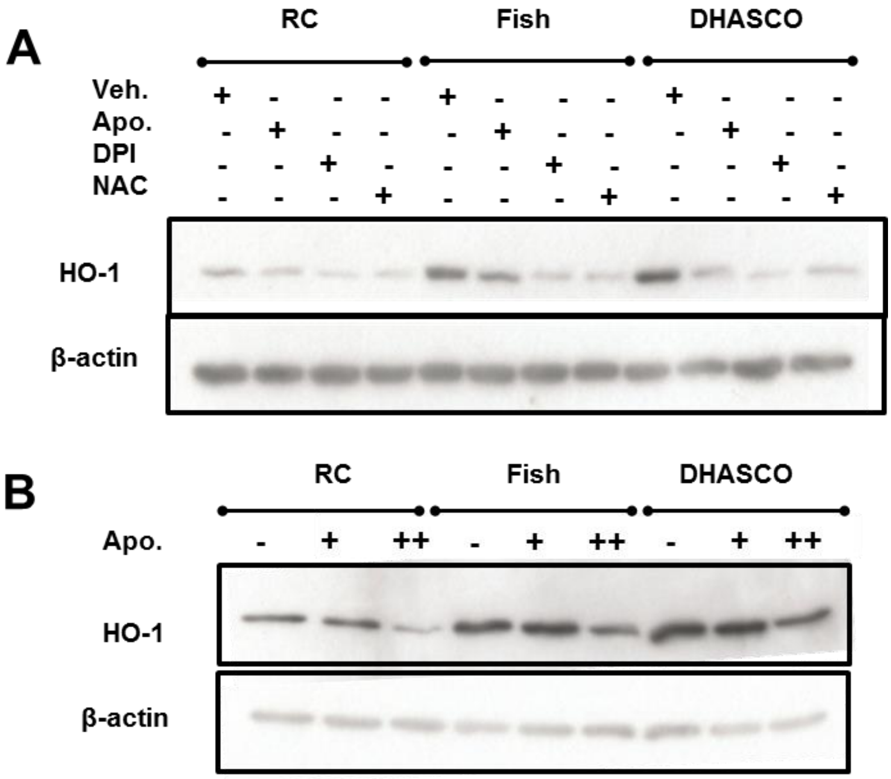

Supplementary Figure 2: Effects of anti-oxidants and pharmacological NOX inhibitors on A-CRLP-induced HO-1 expression

HUVEC were cultured in serum-depleted medium (1% FBS) for 1 hour. Cells were pre-incubated (30 min) with vehicle or inhibitor(s) as indicated and then exposed to remnant control (RC), fish or DHASCO® A-CRLPs (280 µM TG) for 4 hours in the continued presence or absence of inhibitor(s). HO-1 and β-actin blots are each representative of 3 independent experiments on 3 individual HUVEC isolates. **A.** Veh - DMSO; Apo - apocynin (1 µM); DPI - diphenyleneiodonium (20 µM); NAC - *N*-acetylcysteine (5 mM). **B.** DMSO vehicle (-); 100 µM apocynin (+); 1 mM apocynin (++).

Supplementary Figure 3

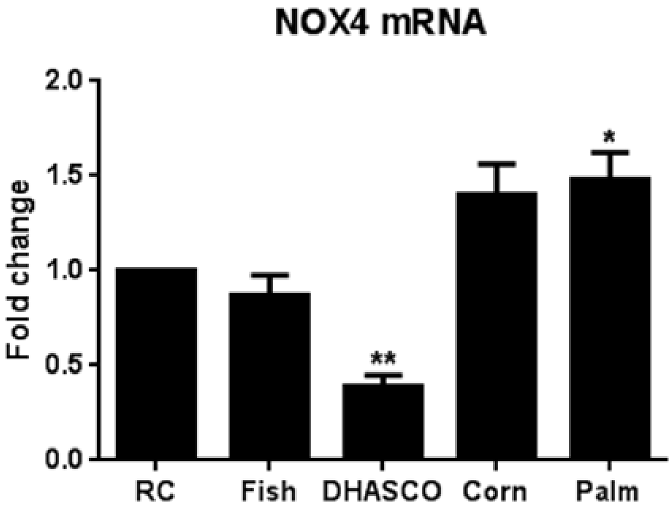

Supplementary Figure 3: Effects of A-CRLPs on NOX4 expression in aortic ECs

HAECs were incubated with A-CRLPs as indicated (280 µM TG) for 16 hours and NOX4 levels assessed by qPCR and normalized to GAPDH. Data are mean fold change relative to remnant control (RC) ± SEM (n = 5). *P < 0.05, **P < 0.01 (One-way ANOVA with Dunnett's multiple comparisons test *versus* RC).

Supplementary Figure 4

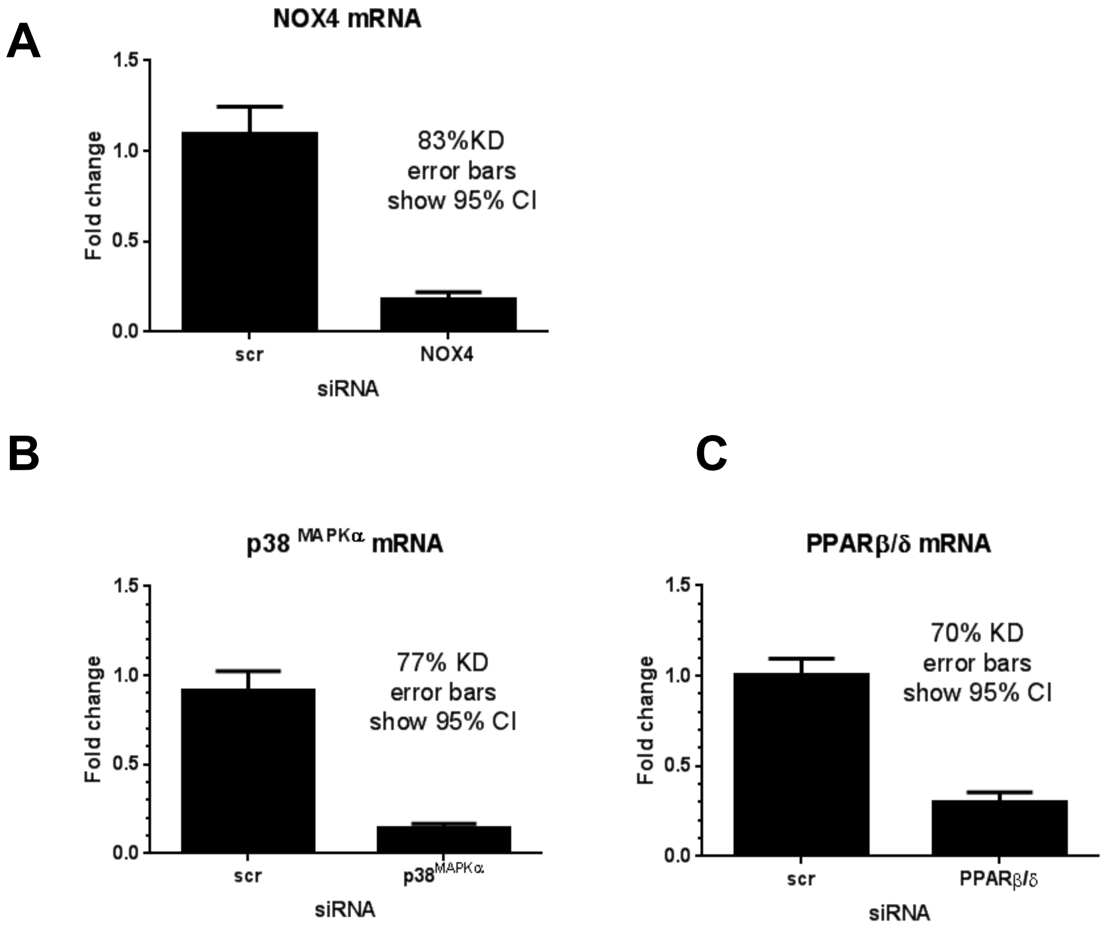

Supplementary Figure 4: Confirmation of gene knockdown in HAEC using siRNA

HAECs were treated with scrambled siRNA or with siRNAs targeting NOX4 (**A**), p38$^{MAPK\alpha}$ (**B**) or PPARβ/δ (**C**). Cells were then analyzed for mRNA expression and data normalized to GAPDH. Results are presented as fold change relative to RC ± 95 % CI; n = 3 (**A**), n = 4 (**B** and **C**). KD = knockdown efficiency.

Supplementary Figure 5

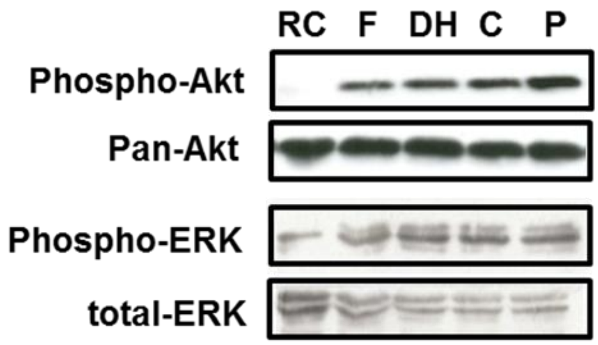

Supplementary Figure 5: A-CRLPs activate Akt and ERK1/2 in HAEC regardless of TG composition

Serum-starved HAEC were incubated with remnant control (RC), fish (F), DHASCO® (DH), corn (C) or palm (P) A-CRLPs at 280 µM TG for 10 minutes and the phosphorylation states of Akt and ERK1/2 measured by western blotting using phospho-specific antibodies. Representative blots from 3 independent experiments are shown.

Supplementary Figure 6

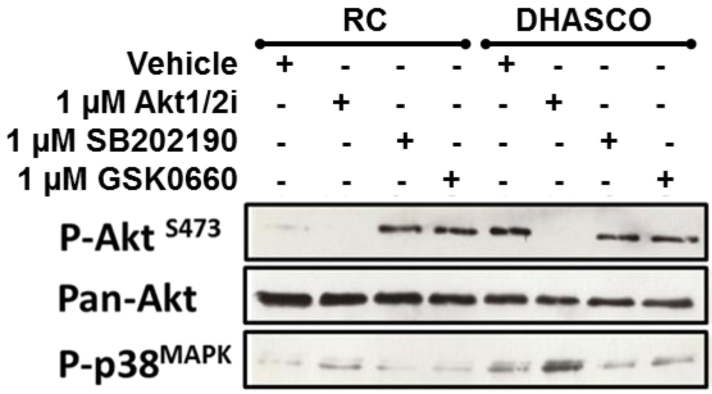

Supplementary Figure 6: Confirmation of inhibitory effects of pharmacological kinase inhibitors

HUVEC were incubated for 1 hour in serum-depleted medium (1% FBS) and then incubated for 30 minutes with DMSO vehicle, SB202190 ($p38^{MAPK}$ inhibitor) or GSK0660 (PPARβ/δ antagonist) at the indicated concentrations. Cells were then incubated with remnant control (RC) or DHASCO® A-CRLPs (280 µM TG) for 10 minutes in the continued presence or absence of inhibitor. Western blots for phospho (P)-Akt, pan-Akt (loading control) and phospho-$p38^{MAPK}$ are shown.

Supplementary Figure 7

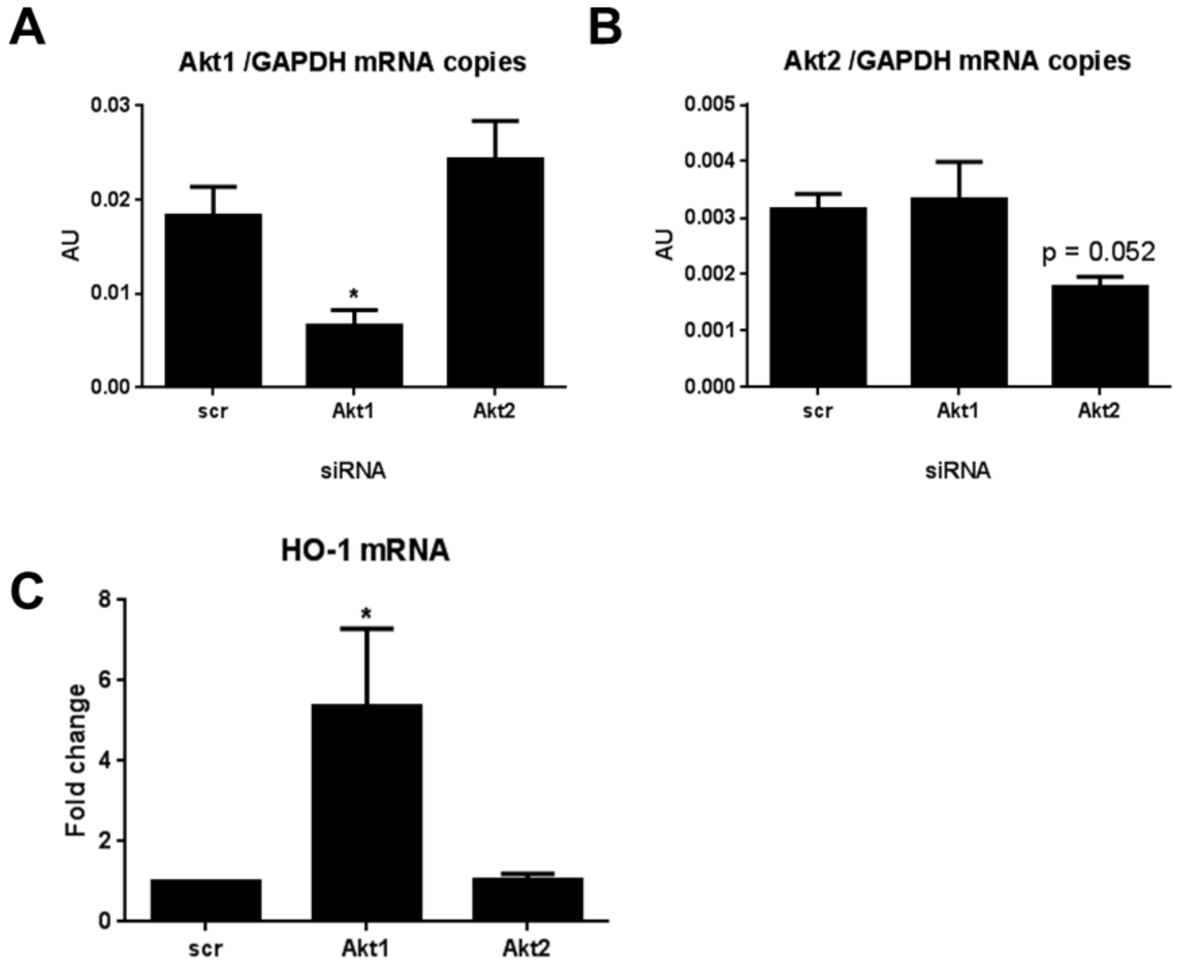

Supplementary Figure 7: Selective knockdown of Akt isoforms by siRNA and effects on HO-1 expression in unstimulated cells

HAECs were treated with scrambled non-coding control (scr) siRNA or with siRNAs targeting Akt1 or Akt2. Expression of mRNAs for Akt1 (**A**), Akt2 (**B**) and HO-1 (**C**) was analysed by qPCR and normalized to GAPDH. Data are mean ± SEM (n = 4 independent experiments). Akt1 is the predominant isoform expressed in HAEC (~0.02 Akt1/GAPDH mRNA copies). Comparatively low levels of Akt2 were present (~0.002 Akt2/GAPDH mRNA copies) and Akt2 was not detectable using immunoblotting (not shown). *$P < 0.05$ (One-way ANOVA with Dunnett's multiple comparisons test).
